# Cross-species engraftment biases and metabolic divergence in gnotobiotic mice humanized with ulcerative colitis microbiota

**DOI:** 10.1080/19490976.2025.2581445

**Published:** 2025-11-24

**Authors:** Martina A. Guggeis, Nadia Andrea Andreani, Víctor A. López-Agudelo, Florian Tran, A. Samer Kadibalban, Karlis Arturs Moors, Georgios Marinos, Abdulgawaad Saboukh, Danielle Harris, Maren Falk-Paulsen, Saskia Weber-Stiehl, Lea Järke, Felix Sommer, Lina Welz, Corinna Bang, Andre Franke, Cecilia J. Chung, Christina Bronowski, Sven Schuchardt, Sven Künzel, Konrad Aden, Stefan Schreiber, Christoph Kaleta, John F. Baines, Philip Rosenstiel

**Affiliations:** a Institute of Clinical Molecular Biology, Christian-Albrechts-University Kiel and University Hospital Schleswig-Holstein Campus Kiel, Kiel, Germany; b Department of Internal Medicine I, University Hospital Schleswig-Holstein Campus Kiel, Kiel, Germany; c Max Planck Institute for Evolutionary Biology, Plön, Germany; d Department of Biology and Biotechnology "Charles Darwin", Sapienza University of Rome, Italy; e Research Group Medical Systems Biology, Institute for Experimental Medicine, Kiel University, Kiel, Germany; f Institute of Clinical Chemistry, University Hospital Schleswig-Holstein Campus Kiel, Kiel, Germany; g CAU Innovation GmbH, Kiel University, Schleswig-Holstein, Kiel, Germany; h Section of Evolutionary Medicine, Institute for Experimental Medicine, Kiel University, Kiel, Germany; i NYU Grossman School of Medicine, New York University, New York, USA; j Department of Clinical Infection, Microbiology and Immunology, Institute of Infection, Veterinary & Ecological Sciences, University of Liverpool, UK; k Fraunhofer-Institut für Toxikologie und Experimentelle Medizin, Hannover, Germany

**Keywords:** Microbiome, fecal microbiota transfer, metabolites, IBD, gnotobiotic mice, ulcerative colitis, multi-omics

## Abstract

Ulcerative colitis (UC) is a chronic inflammatory disease of the human colon. Dysbiotic gut microbiota play a central role in its pathogenesis, and alterations in microbial composition and function are closely linked to disease activity. Humanized gnotobiotic mice are increasingly used to study how dysbiotic, human-derived microbial communities shape intestinal inflammation. However, the fidelity of microbiota engraftment and its impact on host physiology and metabolism remain incompletely understood. In this study, we performed a multiomics analysis following fecal microbiota transfer (FMT) from eight patients with active UC into germ-free C57BL/6N mice (five mice per donor). The mice were monitored over three weeks. Longitudinal analysis of microbial communities was performed using 16S rRNA (bacteria) and ITS2 (fungi) amplicon sequencing. Microbial metabolic flux was inferred via genome-scale metabolic modeling, and plasma metabolites were assessed by targeted metabolomics. We observed donor-specific physiological changes in recipient mice, including variations in body weight and adipose tissue. Spontaneous colonic inflammation occurred in one group and was subsequently linked to unintended transfer of *Clostridioides difficile*, which was previously clinically unrecognised in the donor. While bacterial engraftment overall was generally donor-specific and stable across mice, fungal taxa were transferred inconsistently and at low abundance. Despite similar overall plasma metabolomic profiles, select metabolites, including 3-indoleacetic acid, were differentially associated with specific microbial taxa. Moreover, metabolic modeling revealed disrupted metabolic exchange networks in the mouse microbiota compared to the original human donor communities. In conclusion, while human FMT into germ-free mice reliably transmits bacterial features, it introduces metabolic alterations and fails to fully reproduce the fungal microbiome. These findings underscore the need for cautious interpretation of microbiota-driven effects in gnotobiotic models and highlight the limitations of current approaches in replicating the full complexity of human gut ecosystems.

## Introduction

Inflammatory bowel diseases (IBDs), including Crohn’s disease (CD) and ulcerative colitis (UC), are chronic disorders of the gastrointestinal tract that lead to significant morbidity and long-term complications, including hospitalization and surgery.^1^^,^^2^ The prevalence of IBD is increasing worldwide, with a notable rise in developing countries.^3^ The pathophysiology of IBD is multifactorial, involving genetic, environmental, and microbial factors, yet the exact mechanisms driving disease remain poorly understood.^4^^‐^^6^

Several studies have shown that the gut microbiota of IBD patients is dysbiotic, exhibiting altered bacterial diversity and composition compared to healthy individuals.^7^^,^^8^ As a result, the microbiota's functions, metabolite production, and metabolic interactions with the human host also differ from those observed in healthy individuals,^9^^‐^^12^ which has been linked to altered immune responses.^13^^‐^^17^ We have further demonstrated shifts in the metabolic exchange of gut microbiota in IBD patients before and after anti-TNF treatment, as well as distinct differences between responders and non-responders to the therapy.^18^ Although most microbiota studies have focused on bacterial communities, growing interest is now being placed on the role of fungi in shaping metabolic and immune responses in the context of IBD pathogenesis.^19^^,^^20^ Gaining deeper functional insights into these complex host-microbiota interactions in the gut could pave the way for more targeted diagnostic and therapeutic strategies in IBD.

Human studies aiming to address the causality of microbial factors are challenging due to countless extrinsic and intrinsic confounding factors such as diet, medication, age, comorbidities, or genetics on the microbiome and its metabolites. To overcome these limitations, investigations in germ-free (GF) animals offer a powerful platform for dissecting the mechanistic effects of the microbiota on the host immune system and gut. Through the transfer of individual strains or more complex microbial communities, gnotobiotic mouse models enable the controlled investigation of microbes and their metabolites on the host.^21^^,^^22^ A striking example of gnotobiotic mouse models in IBD research is the study by Nagao-Kitamoto et al. (2016), which showed that, compared with those given stool from healthy controls, mice receiving stool from CD patients presented altered mucosal gene expression. Furthermore, transplanting stool from these humanized mice into IL-10-KO colitis-prone mice revealed increased colitis susceptibility in those receiving CD-derived microbiota.^23^ Sheikh *et al.* further confirmed that colitis can be induced in germ-free mice inoculated with stool from CD patients even without genetic or chemical induction of colitis.^24^ Given the significant potential of microbiome transfer models in mice for studying host‒microbiome interactions in IBD, further characterization is essential – particularly to assess the efficiency of microbial feature transfer and its impact on the host.

In this study, we conducted a structured multiomics analysis (16S rRNA gene and ITS2 amplicon sequencing and targeted plasma metabolomics) of mice transplanted with fecal microbiota from eight UC donors with varying disease activity, using a case-only design. We first analyzed whether the transfer induced an inflammatory phenotype in the mice and whether the level of inflammation in individual donors was associated with this phenotype. We then described the heterogeneity of engraftment of bacteria and fungi in the recipient mice within the donor groups and compared them across donors. We employed metabolic modeling to predict interactions within the bacterial community and assess how these functions differ between mice and humans. In order to explore whether donor effects extend beyond the gut, we also performed novel profiling of the lung and skin microbiomes in the same recipient mice. Finally, we analyzed differences in the plasma metabolome of recipient mice across donor groups and linked specific metabolite features to bacterial taxa.

## Materials and methods

### Stool sample collection and preparation

Ethical approval from the Medical Ethical Commission of the Christian-Albrechts-Universität Kiel was obtained for the collection and use of biological samples (A124/14; AZ 156/03-2/13; A101/20). The patients gave their informed consent for their participation in the study cohort. Stool from eight anti-TNF-naïve patients with UC with acute intestinal inflammation was collected before their planned initiation of treatment with the anti-TNF antibody infliximab. All patients had a disease extent of E2 (left-sided UC) or E3 (pancolitis) according to the Montreal classification. Colonic inflammation was quantified using the Mayo score.^25^ Parts of the study data were collected and managed using REDCap electronic data capture tools.^26^^,^^27^ Stool was collected from the patients, frozen, and stored at -80 °C until preparation for fecal transfer. On the day of gavage, the samples were thawed, and each sample was diluted in sterile prereduced PBS (1:10 w/v) under anaerobic conditions. After brief vortexing, the samples were passed through a 100 mm cell strainer, and 100 µL of the solution was used for gavage per mouse, with a total of five mice per donor sample.

### Experimental animals

The study was performed according to approved animal protocols and institutional guidelines of the Max Planck Institute for Evolutionary Biology, Plön. The mice were maintained and handled in accordance with FELASA guidelines and German animal welfare law (Tierschutzgesetz § 11, permit from Veterinäramt Kreis Plön: 1401-144/PLÖ-004697), and the experiments were performed in accordance with FELASA guidelines and German animal welfare law § 8 permit from the “Ministerium für Landwirtschaft, ländliche Räume, Europa und Verbraucherschutz” (MLLEV) 107-11/18. GF C57BL/6NTac male mice aged approximately 10 weeks (*n* = 40) were ordered from Taconic Biosciences. The mice were held in isolators (Class Biologically Clean, Wisconsin, USA) for 2 weeks after arrival for acclimatization under sterile conditions.^28^^,^^29^ The mice were held under a 12 h light/12 h dark cycle at 22 °C ( + /− 2 °C) and a humidity of 55%−60%. The mice had access to V1124-927 mouse breeding food sterilized at 50 kGy (Sniff, Soest, Germany) and sterile water *ad libitum*.

The mice were aseptically extracted from the isolator and handled under a sterile flow hood for weighing and stool sample collection at the beginning of the experiment. Five mice for each donor were then colonized following established procedures^23,30^^‐^^33^ by gavage with 100 µL of the previously processed human fecal material using a 18 G reusable feeding needle (Fine Science Tools, Heidelberg, Germany) before being transferred into individual IsoCages (Tecniplast, Italy). Briefly, stool preparation was performed under anaerobic conditions by weighing 300 mg of the feces and diluting it in 3 mL (1:10 w/V) sterile prereduced PBS. The samples were vortexed and passed through a 100 µm cell strainer. The prepared samples were used for gavage (100 µL per mouse). The mice were extracted from the cages under aseptic conditions once a week for weighing and fecal sample collection. The sterile flow hood and all materials were disinfected between the handling of each mouse group. After three weeks, the mice were sacrificed for tissue collection.

### Tissue collection and histological assessment

Mice were sacrificed using CO_2_. Heart puncture was performed to collect blood, which was transferred to a lithium-heparin tube and centrifuged to obtain plasma, which was stored at -80 °C. Abdominal organs were extracted and weighed, with organ weights being normalized to a percentage of the mouse’s total body weight. The length of the small intestine and colon was measured prior to flushing with PBS to remove the luminal contents. The small intestine and colon were then cut longitudinally, arranged in a Swiss roll and fixed in 10% formalin for histological assessment.

Formalin-fixated colonic and small-intestinal tissue was stained with H&E. Histological scoring was performed in a blinded manner as described elsewhere.^34^

### Fecal lipocalin−2 measurement

Feces were diluted in 1.5 ml sterile prereduced PBS and homogenized for 30 min at maximum speed on a horizontal vortex (Vortex Mixer Model Vortex-Genie® 2, Scientific Industries, Bohemia, NY, USA). The samples were then centrifuged for 5 min at 10,000  rpm before the supernatant was collected for lipocalin-2 measurement using the commercial kit Mouse Lipocalin−2/NGAL DuoSet ELISA (R& D Systems, Minneapolis, MN, USA) for mouse Lipocalin-2 (DY1857) as described elsewhere.^35^ Testing was performed following the manufacturer’s instructions. The samples were diluted 1:10 and added to the plate. The optical density of each well was determined with a plate reader (SPARK, Tecan, Tecan, Männedorf, Switzerland) at 450 nm and 540 nm. Lipocalin-2 concentrations were normalized by the weight of feces for each sample.

### Targeted metabolomics

Plasma and serum metabolomics was performed using the Biocrates MxP® Quant 500 Metabolomics Kit (Biocrates Life Sciences AG, Innsbruck, Austria) according to the manufacturer’s instructions using chromatography tandem mass spectrometry (LC–MS–MS). The kit analyses 630 metabolites using a QTRAP 5500 SCIEX mass spectrometer (SCIEX, Darmstadt, Germany) and an Agilent 1290 Infinity II liquid chromatography system (Agilent Technologies, Santa Clara, United States), and the analyses were performed as described elsewhere.^36^ A modified 80% rule^37^ was applied using a cutoff of 75% per group, meaning that the metabolites were retained if they had at least 75% values above the lower limit of detection (LOD) and of quantitation (LLOQ) in at least one donor group of recipient mice. This cutoff was selected because most of the mouse groups had four surviving mice, indicating the quantifiable presence of the metabolite in three-quarters of the mouse groups. The values below the LOD and LOQ were then replaced by the LOD divided by two and the LLOQ divided by two, respectively.

### 16S rRNA gene amplicon sequencing

Fecal DNA was extracted from human and mouse feces using the QIAmp PowerFecal Pro DNA Kit (Qiagen) according to the manufacturer’s instructions. Additionally, RNA was extracted from mouse lung and skin tissue samples using the Qiagen AllPrep DNA/RNA Mini Kit according to the manufacturer's instructions. The RNA was reverse-transcribed using the High-Capacity cDNA Reverse Transcription Kit (Thermo Fisher Scientific) according to the manufacturer's protocol.

#### V3–V4 region sequencing for the fecal samples

The V3–V4 region of the 16S rRNA gene was amplified using 515F and 806 R and sequenced on an Illumina MiSeq sequencer (300PE) for the human and murine fecal samples. Using a dual barcoding approach, we allowed only barcodes without mismatches during the demultiplexing process (Casava, Illumina). Raw 16S rRNA gene sequencing data were processed using the DADA2 pipeline, implemented in the DADA2 R package, version 1.16.0.^38^ Specifically, the first 20 bases were trimmed from the 5’ end of forward or reverse read sequences, the 290 bases from the 3’ end of forward or reverse sequences were truncated, and the reads were also truncated at the first instance of a quality score smaller than or equal to 3. An abundance table of 16S rRNA amplicon sequence variants (ASVs) was generated, and the taxonomic annotation of ASVs was obtained using the SILVA 138 database.^39^ Individual data sets were randomly subsampled to 6,500 reads as described to normalize the sequencing depth.

#### V1–V2 region sequencing for fecal and tissue samples

The V1–V2 hypervariable region of the 16S rRNA gene is often preferred for investigating microbial communities in low-biomass samples, such as the skin and lung, owing to its relatively high sensitivity.^40^^,^^41^ Thus, sequencing was performed on the extracted DNA and reverse-transcribed RNA (cDNA) from lung and ear skin tissues, the Zymo mock community, and the DNA from fecal samples of human donors and mice to allow comparison with previous samples. The V1–V2 hypervariable region of the 16S rRNA gene was amplified using barcoded primers (27F-338R) following the protocol by Rausch et al.^42^ The resulting amplicons were sequenced with 250 bp paired-end reads on the Illumina MiSeq platform.

#### Data processing

Sequence processing and taxonomy assignment were performed as previously reported, with minor changes: the first 5 bases were trimmed from the 5' end of forward or reverse read sequences, 230 bases from the 3' end of forward or 180 cases from the 3' end of reverse sequences were truncated, and the reads were also truncated at the first instance of a quality score smaller than or equal to 2. The prevalence method Decontam package version 3.19.0^43^ was employed to computationally identify potential contaminants based on the microbial composition of both the true samples and the negative controls. The phyloseq R package, version 1.48.0,^44^ was employed for data management and analysis. The analysis was conducted on lung, skin, and a combination of all bodily sites (mouse lung, mouse skin, and mouse feces), as well as the human fecal samples. As the analysis was performed separately for each body site, rarefaction was applied independently to each tissue type, resulting in distinct sequencing depths: mouse skin (10,000 reads), mouse lung (200 reads), mouse feces (1,100 reads), and human fecal samples (6,200 reads).

### Quantitative polymerase chain reaction (qPCR)

The presence or absence of *Clostridioides difficile* toxins A and B and binary toxins A and B was assessed using qPCR. Fecal bacterial DNA from recipient mice one week after microbial transfer and from the donor samples was first diluted to a working concentration of 4 ng per well in nuclease-free water to a final volume of 150 μL or, if this was not possible, to the highest achievable concentration. For each sample, 5  μl of DNA was added to a well in technical duplicate with 0.5  μl of the primer mixture (5  μM). Then, 4.5 μl of SYBR Select Mastermix (Thermo Scientific) was added. Water was used as a negative control. The plate was sealed with foil, briefly vortexed and centrifuged. DNA quantification was performed in a QuantStudio 7 Flex Real-Time PCR System (Thermo Scientific) with the following cycle: 2 min at 50 °C, 10 min at 95 °C, followed by 45 cycles of 95 °C for 15  s and 60 °C for 1 min. The 16S rRNA gene with primers 338 forward and 805 reverse was used as a positive control for bacterial DNA in all samples, while *C. difficile* specific *tpi* encoding triose phosphate isomerase was used to detect the presence of *C. difficile* in the samples, using the primer described by Lemee et al.^45^ Furthermore, primers for *tcdA, tcdB* encoding toxins A and B, *cdtA* and *cdtB* encoding binary toxins A and B were used to assess additional risk for intestinal adhesion using primers described by Wroblewski et al.^46^ Primer sequences are provided in Supplementary Table 1.

### Metabolic modeling

#### Mapping and metabolic model construction

The fecal 16S rRNA gene sequences were mapped against a set of 5,416 human gut bacteria from the HRGM collection^47^ using the Usearch tool.^48^ The mapping identified 284 (49%) bacterial species from the human donors and 231 (22%) of the colonized germ-free mice. We thereafter used gapseq^49^ for the reconstruction of genome-scale metabolic models for each of the identified gut bacterial strains, with these models being compatible with the sybil R package model formats.^50^ We used the standardized mouse diet (ssniff) for the mouse samples^51^ and the standard North European diet as described previously^52^ for the human samples as nutritive inputs for inferring genome-scale metabolic models.

#### Community models and coupling approach

For each of the microbial communities that was collected from the fecal samples, we reconstructed community metabolic models using flux balance analysis (FBA) with a coupling approach implemented in the MicrobiomeGS2 R package.^53^ First, we estimated individual bacterial growth using FBA by setting the growth reaction as an objective function and solving the model. We excluded 29 bacterial models with low estimated growth rates (<10⁻³), as they can drastically reduce the overall growth of the community. We then joined the bacterial models belonging to the same community in a merged model, where each model has its own compartment, and all the models were connected through an environmental compartment to freely exchange metabolites. The microbial abundances were used as coupling constraints to build the community model.^52^ We set the following coupling constraints: c = 400 mmol.gDw^−1^.hr^−1^, as originally proposed, and a more relaxed coupling u = 1e−6 mmol.gDw−1.hr−1. An objective function was set to optimize community growth with concomitant minimization of total flux. The cplexAPI R package^54^^,^^55^ was thereafter used to run the metabolic simulations using the ILOG CPLEX Optimization Studio solver V12.1 with an academic license.^56^

### Internal transcribed spacer 2 (ITS2) genomic region gene sequencing

For sequencing of the ITS2 region and for library preparation, fecal DNA extracted as described above was sent to the Microbiome Laboratory of the Competence Centre for Genomic Analysis Kiel (CCGA). PCR and sequencing were performed with the primer pair 5.8S-Fun and ITS4-Fun as described elsewhere^57^using an Illumina MiSeq machine (250 paired-end). Denoising was performed with the DADA2 R package, with default parameters applied unless otherwise stated. The reads were truncated at the first base where the quality score dropped below *Q* = 5, the maximum number of mismatches in the overlap region was 4, and the minimum lengths of the reads after truncation were 230 bp and 150 bp for forward and reverse reads, respectively. Taxonomic annotation was attributed to the obtained ASVs using the UNITE database (v 9.0).^58^ Decontamination of the sequenced reads was performed using the decontam R package with the prevalence method using a cutoff of 0.2.^43^ Sequencing was unsuccessful for 9 of the murine samples across the three sampling timepoints after microbiome transfer.

### Statistical analysis

Statistical analysis was performed using R software version 4.3.0^59^ in RStudio version 2023.6.0.421.^60^ Comparisons between the eight donor groups were performed using Kruskal-Wallis test with a significance cutoff of alpha = 0.05. When several features, such as mouse features, were compared between groups, Benjamini‒Hochberg (BH) correction was applied to the Kruskal‒Wallis results. In the case of significant differences between groups, Dunn’s test for pairwise multiple comparisons was used followed by BH correction with a cutoff of alpha = 0.05. Metabolic modeling data was normalized by dividing it by the predicted growth prior to analysis unless stated otherwise.

Diversity measures in the 16S rRNA gene data and metabolic modeling data such as Shannon diversity and Bray‒Curtis diversity were computed using the vegan package version 2.6.4,^61^ and principal coordinates were extracted using the ecodist package.^62^ Principal component analysis for the metabolomics data was performed using the prcomp function, and Euclidean distances were calculated using the dist function from the R stats package.^59^ Distance measures were compared between groups using the Permutational analysis of variance (PERMANOVA) in the vegan package. The multivariate homogeneity of group dispersions (variances) between data from human and mouse samples was calculated using the betadisper function implemented in vegan. When comparing the contaminated and noncontaminated mouse groups, nested ANOVA accounting for the group factor was applied.

To explore the variation between and within donor groups per time at the genus level, the intraclass correlation coefficient (ICC) was computed via the *ICCest* function of the ICC R package (v.2.4.0),^63^ which uses the longitudinal rarefied abundances as input, defining each individual donor group as a distinct class. ICC estimation uses the variance components from a one-way ANOVA (between-group variance and within-group variance; $\mathrm{ICC}=\mathrm{va}{\text{r}}_{\mathrm{between}}/(\mathrm{va}{\text{r}}_{\mathrm{between}}+\mathrm{va}{\text{r}}_{\mathrm{within}})$). Similarly, the between-individual and within-individual coefficients of variation were computed for each genus: $\mathrm{CV}=\frac{\text{σ}}{\mu }$. The between-individual coefficient of variation was computed for all timepoints or for each time point across different individual donor groups, and the within-individual coefficient of variation was computed for each individual donor group across time points.

To understand the major sources of variation attributable to the donor group in the multitissue and multiomics layer data (gut, skin, and lung microbiome, plasma metabolomics and metabolic modeling), we applied variance partitioning (v.1.26.0),^64^ which uses linear mixed models to compute the attributable percentage of variation of a feature based on selected covariates. Here, we selected the donor group as the main contributor to the variation in the gut, skin and lung microbiota composition; plasma metabolomics; and metabolic flux exchange within the gut bacterial communities.

Correlations were performed using Spearman correlations, whereas the coin package version 1.4-3 was used with 9999 permutations, with the distribution setting “approximate” and ties method “average scores”.^65^ For correlations of the individual metabolic fluxes with mouse features, partial Spearman correlations correcting for growth using the nonnormalized fluxes were performed using the ppcor package.^66^ For correlations with 16S rRNA gene data, only genera that had five counts or more in one sample and nonzero counts in at least three samples were included. For metabolic modeling, only fluxes with at least three unique values were retained and used for correlation. The weight differences at week 1 and week 2 were correlated with the 16S rRNA gene data and metabolic modeling data from week 1 and week 2, respectively, while all other mouse physiological variables that were collected at week 3 were correlated with the data from week 3. Associations between donor features and individual bacterial ASVs and metabolic fluxes were computed using linear mixed-effects models with group as a random effect using the lme4 package,^67^ with *p*-values computed using the lmerTest package.^68^ All correlation analyses were corrected for multiple testing using BH-correction. Simple pairwise comparisons were performed using unpaired Wilcoxon test followed by BH correction for multiple comparisons.

## Results

### Donor characteristics and fecal microbiota transfer

Eight anti-TNF-α-naïve UC patients with moderate to severe levels of inflammatory activity were selected for the experiment, with a total Mayo score ranging from 6 to 11 points (Table 1). Patients received treatment with either oral or topical corticosteroids and/or 5-aminosalicylic acid and/or azathioprine, with no other concurrent immunosuppressive therapies at the time of stool sampling (Supplementary Table 16).

**Table 1.** Patient characteristics at time of stool sampling.

Characteristics	Patients (*N *= 8)
Age in years (Mean ± *SD*)	32.1 ± 9.6
Males/females (*n* [%])	5/3 [62.5/37.5]
BMI in kg/m^2^ (Mean ± *SD*)	21.3 ± 3.5
Total Mayo score (Median [IQR])	8.5 [7.0−9.25]
Partial Mayo score	6 [4.75−6.25]
Endoscopic Mayo score	3 [2.75−3.0]
C-reactive protein in mg/L (Mean ± *SD*)	10.36 ± 1.51
Blood Leukocyte count in 10^9^/L (Mean ± *SD*)	8.94 ± 3.41

### Physiological consequences of FMT from different UC donors to germ-free mice

**Figure 1.**
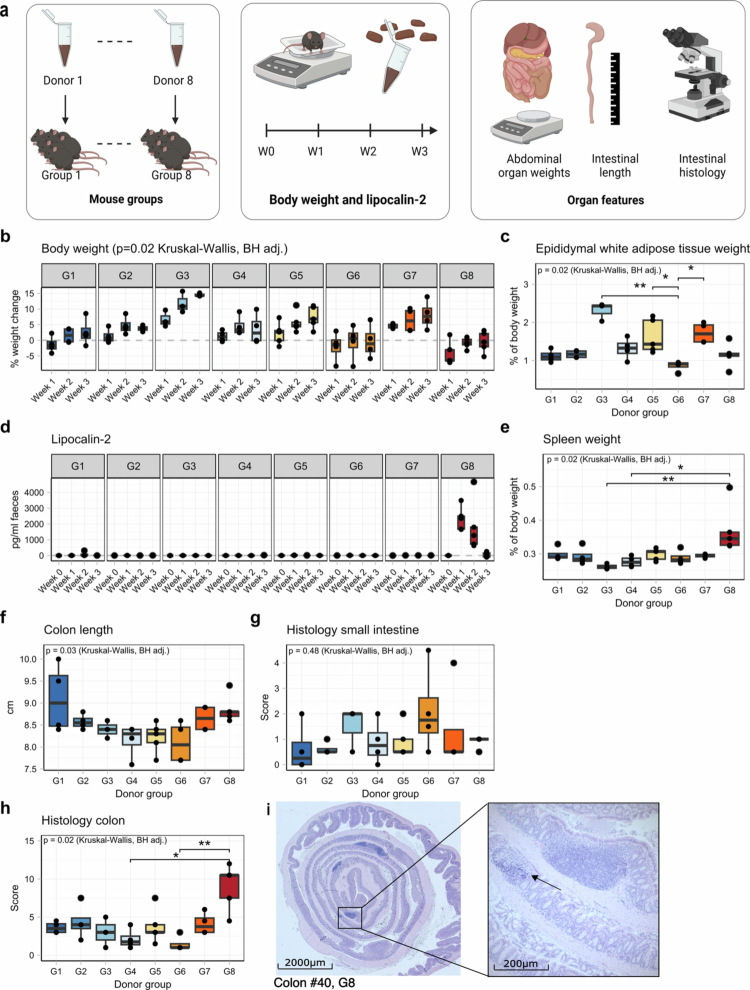
Physiological effects of FMT from different UC donors in germ-free mice. (a) Visual representation of the experimental set-up. The mice were divided into eight groups of five mice, and each group was gavaged with the stool sample of one patient with ulcerative colitis, with 33 mice completing the experiment until the end of week 3. (b) Percentage body weight change of the mice recorded longitudinally per donor group. (c) Epidydimal white adipose weight as a percentage of total body weight per donor group. (d) Longitudinal fecal lipocalin-2 levels per donor group. (e) Spleen weight as a percentage of total body weight per donor group. (f) Absolute colon length in cm per donor group. (g, h) Histological inflammatory score of (g) the small intestine or (h) the colon per donor group. (i) Representative colonic histology with H&E staining of a mouse in group 8. Inflammatory cell infiltration with epithelial erosion is indicated by the arrow. (*:*p* < 0.05; **:*p* < 0.01; ***:*p* < 0.001; Dunn’s test with Benjamini‒Hochberg correction).

We evaluated a panel of physiological parameters after microbiota transfer from individual UC donors to investigate how each donor's microbiome influences host health both over time (weight) and at the end of the three-week postgavage period (colon length, epididymal white adipose tissue (EWAT) and spleen weight, small intestinal and colon histology, Figure 1a). Among the 40 mice that entered the experiment, seven were euthanized after reaching a predefined humane endpoint, primarily due to signs of systemic infection, with no significant differences observed between the experimental groups. The total body weight changes were strikingly different among the groups at all three time points, with the greatest differences occurring at week 1 after gavage (*p* = 0.016; Figure 1b). The weight gain of the mice in donor group 3 (G3) was the greatest at all timepoints, with an average weight of 14.5% greater than their original body weight after three weeks, while mice in G8 was on average 4.2% of their weight one week after gavage, and these mice only slowly regained weight in the following 3 weeks (Figure 1b). EWAT relative weight was significantly different between the groups after correction for total body weight (*p* = 0.016, Figure 1c). Fecal lipocalin-2 concentrations significantly differed between groups at week 1 and week 2 (*p* = 0.012 and *p* = 0.0003, respectively), with a significantly higher concentration in the mice of G8 than in those of G3 and G4 at week 1 and those of G2-G4 at week 2 (BH-corrected *p* < 0.05). The other groups showed minimal or no increase (Figure 1d). The change in lipocalin levels and marked weight loss, along with the increased spleen weight (Figure 1e), suggest that FMT triggered an inflammatory response in G8, distinguishing them from the other groups. While there was significant variation in colon length between the groups overall (*p* = 0.033 Kruskal‒Wallis with BH correction), G8 did not significantly differ from the other groups upon pairwise comparison (Figure 1f). However, microscopic analysis confirmed significantly increased histological inflammation scores in the colons of mice from G8 but not in the small intestine (Figure 1g-i, Supplementary Figure 1). The relative cecum weight, relative liver weight, and small intestinal length did not significantly differ between the groups (*p* > 0.05, Kruskal‒Wallis with BH correction, Supplementary Table 2).

Next, we performed a systematic correlation analysis between the clinical data of the donors and the physiological traits of the mice, selecting only features that significantly differed between the groups. Significant positive correlations were detected between the histology scores of the mice and clinical disease activity (full and partial Mayo scores), as were the body mass index (BMI) and leukocyte counts of the donors. However, when G8, which displayed a colitis-like phenotype, was excluded, all associations lost significance, indicating that G8 was an outlier driving the observed correlation effect (Supplementary Figure 2).

### Group- and individual-level variation of bacterial colonization by donor communities in recipient mice

**Figure 2.**
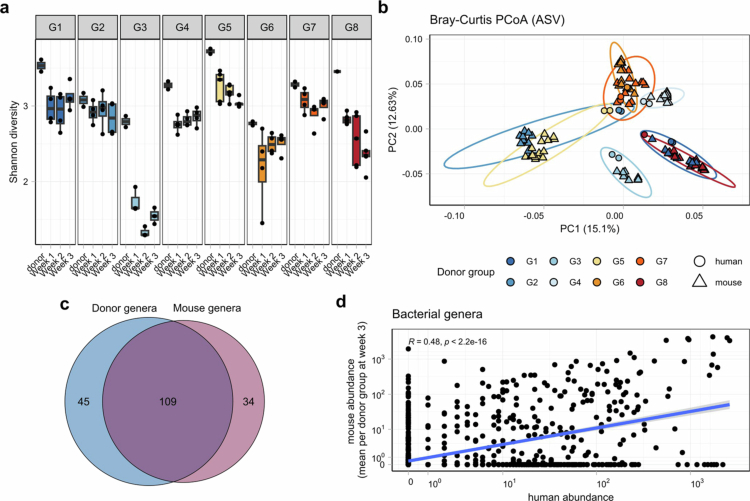
Analysis of group- and individual-level bacterial colonization efficacy. (a) Shannon diversity per donor group, including the donor sample and PBS-diluted sample for gavage, was calculated at the rarefied ASV level. (b) Principal coordinate analysis (PCoA) based on Bray‒Curtis distances was performed on rarefied ASV abundances of human and mouse stool samples per group. The ellipses indicate 95% confidence intervals. (c) Venn diagram of the total number of unique genera present in any human stool sample and any mouse stool sample, unrarefied counts. (d) Scatterplot of rarefied genera abundances of human samples and the mean abundances of their respective recipient mice after 3 weeks, Spearman correlation. The blue line depicts the linear fit, and the gray shading represents the ± standard error.

We next analyzed the colonization patterns of the human bacterial communities in the recipient mice using 16S rRNA gene sequencing of the feces (sampled weekly). The donor fecal samples and the donor fecal slurries used for gavage were sequenced and highly similar in terms of *α* diversity and *β* diversity (Figure 2a-b). Community evenness (measured as the Shannon index) was significantly greater overall in the human donor samples compared to the mouse samples at week 2 and week 3 (Supplementary Figure 3). The PCoA plot based on the Bray‒Curtis dissimilarity index clearly revealed group-dependent clustering of the mouse fecal samples (Figure 2b). Analysis of the dissimilarity within the mouse datasets confirmed that the donor group had the strongest influence on clustering (R^2^ = 0.69, *p* < 0.0001), while the respective timepoint contributed little to the explained variance (R^2^ = 0.03, *p* < 0.0001, Figure 2b). Furthermore, donor samples were significantly closer to their respective recipient mice than to the other transplanted mice (Supplementary Figure 4, *p* < 0.0001). To determine the identity and amount of fecal bacterial genera from the human sample that successfully colonized the mouse gut, unrarefied genus-level counts were used. Among the 154 genera identified in all the donors, 109 (70.8%) were present in at least one mouse (Figure 2c). The rarefied abundances of the human fecal samples overall showed a linear relationship with the mean abundances in their respective recipient mouse samples (*R* = 0.48, *p* < 0.001), indicating that higher abundances in the donor generally translated to higher abundances in the mice. Nevertheless, several genera detected in humans were absent in mice, and vice versa (Figure 2d, Supplementary Table 3). These compounds are unlikely to be contaminants, as they were not present in the mice before gavage. Instead, they likely represent low-abundance taxa in the donors that expanded in the mouse gut.

**Figure 3.**
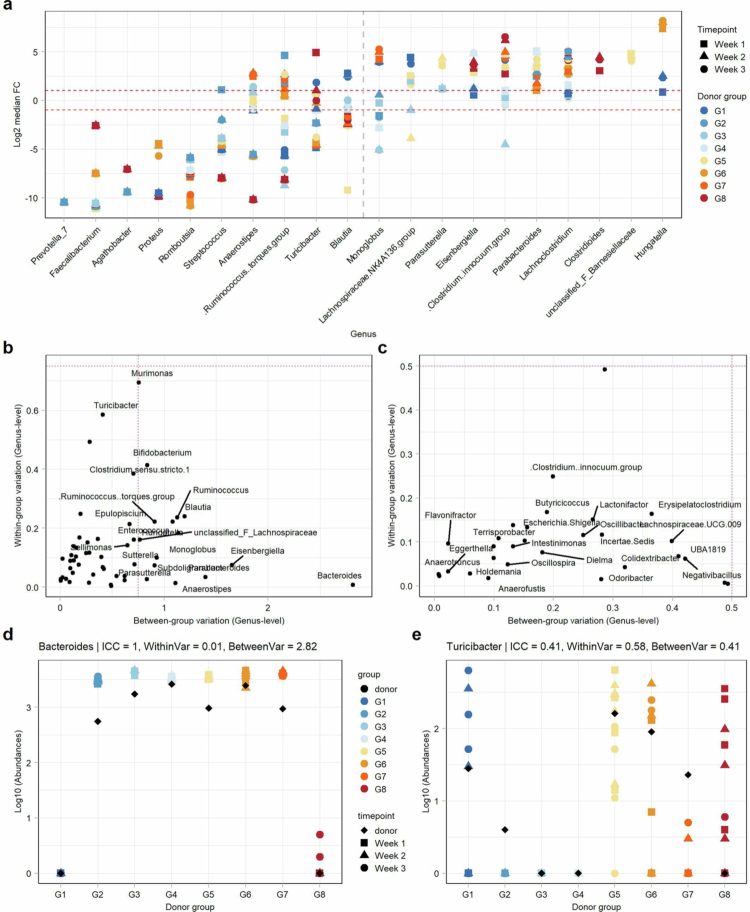
Qualitative bacterial transfer analysis and within- and between-group variation. (a) Log2 median fold change (Log2FC) of abundance in recipient mice relative to the original donor abundance per donor group and per sampling timepoint after transfer. Top 10 bacterial genera with the most negative changes are on the left of the dashed gray line, and those with the top 10 most positive changes are on the right. The dashed red lines at -1 and + 1 Log2FC. (b) Intraclass correlation (ICC) analysis of bacterial genera; labeled genera are those with either a high within-group or between-group variability coefficient of > 0.5. (c) ICC analysis of bacterial genera; labeled genera have a low within-group and between-group variability coefficient of < 0.5. Note the different scales of the horizontal axis. (d-e) Abundance of *Bacteroides* (d) and *Turicibacter* (e) per group in the original donor sample and at three sampling timepoints in recipient mice after microbial transfer.

Among the bacterial genera present in both the donors and the mice, we next explored which showed the greatest changes in relative abundance in the recipient mice compared to the original donor sample. The log-fold change in the mean abundance of the recipient mice revealed that *Hungatella* had the highest relative abundance in mice, while *Prevotella 7* and *Faecalibacterium* were present at lower abundances in the recipient samples than in the donor samples (Figure 3a). Intraclass correlation (ICC) analysis further revealed genera with high or low stochasticity of engraftment and variability within groups, between groups, or both (Figure 3b-c). The genus *Bacteroides* exhibited the highest variability between groups but low variability within each group. Furthermore, a high abundance of *Bacteroides* in the original donor sample also resulted in successful transfer in the recipient mice (Figure 3d). *Turicibacter*, on the other hand, presented high within-group variability with high stochasticity of transfer from donor to mouse. It was among the 10 genera with the greatest negative log-fold change in the mice compared to the donor and showed an incomplete and highly variable transfer in several groups (Figure 3e).

Of note, while the week 0 samples of the mice were expected to be entirely free of bacterial reads, we found varying abundances of ASVs belonging mainly to environmental bacteria from the genera *Bacillus* and *Paenibacillus* in the samples from groups 5−8. Since the animals were sourced from a vendor and consistently tested negative by qPCR upon arrival, this suggests that contamination occurred in this group of germ-free mice within days to hours prior to gavage. There was no indication of contamination by changes in baseline characteristics (e.g., weight shift, lipocalin-2) prior to gavage. Notably, contaminants present during gavage did not persist, as they were completely absent from all the mouse fecal samples from week 1 onwards. Additionally, postgavage *α* diversity did not differ significantly between the contaminated and noncontaminated groups, as shown by an analysis of variance adjusted for group differences. β−Diversity, which was calculated using Bray–Curtis distances, revealed substantial overlap between groups. Although PERMANOVA indicated a significant difference between the contaminated and noncontaminated groups, the explained variance was low. The distribution pattern suggests that *β* diversity differences were driven primarily by group clustering rather than contamination (*p* < 0.0001, *R*^2^ = 0.075, Supplementary Figure 5).

**Figure 4.**
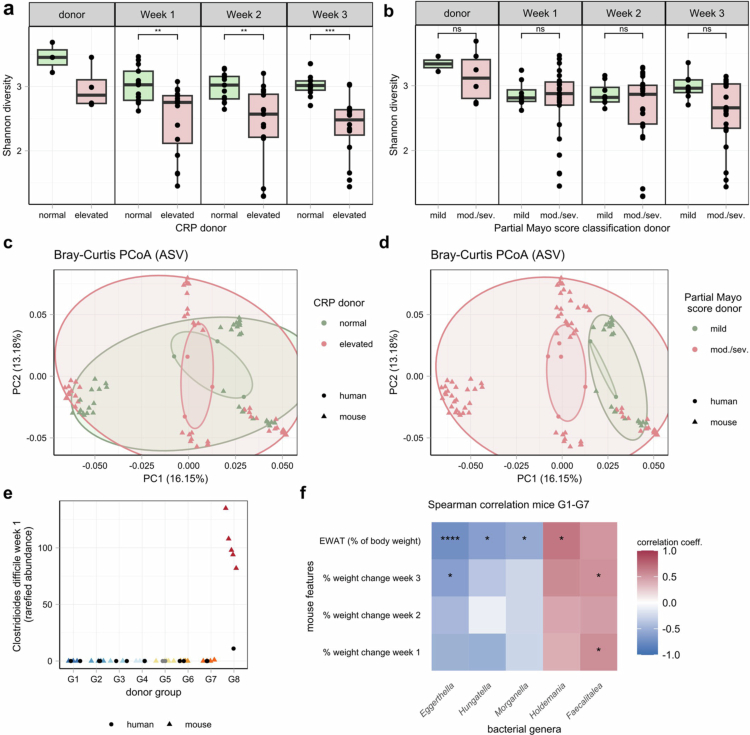
Associations of bacterial features with donor and mouse features. (a, b) Shannon diversity calculated at the rarefied ASV level in the donor and recipient mice at week 1, week 2 and week 3 after transfer. Comparisons between donors with (a) normal (0−5 mg/L) or elevated C-reactive protein (CRP) levels in blood ( > 5 mg/L, data available for 7/8 donors) and the mice of those respective donors, or (b) mild (0−4) or moderate-to-severe (mod./sev.) partial Mayo scores ( > 4). Wilcoxon test with Benjamini‒Hochberg correction. (c, d) Principal coordinate analysis (PCoA) based on the Bray‒Curtis distance of rarefied ASV abundances of human and mouse stool samples, with colours indicating (c) CRP levels of the donor (data on CRP only available for 7/8 donors) or (d) partial Mayo score classification (note the difference between the PCoA plots). (e) Relative abundances of *Clostridioides difficile* one week after microbiome transfer. Two mouse samples (one belonging to G3 and one to G4) did not contain enough DNA for measurements. (f) Spearman correlation of the physiological features and rarefied bacterial genera of the mice in groups 1 to 7, followed by Benjamini‒Hochberg correction. Weight change correlated with the genera sequenced at the respective time points of stool sampling (*: *p* < 0.05; **:*p* < 0.01; ***:*p* < 0.001; ****:*p* < 0.0001). EWAT = epidydimal white adipose tissue.

We next aimed to assess whether differences in the clinical and biochemical inflammatory markers of the donors impacted the composition of the bacterial community in the recipient mice of all the groups, including G8. A comparison of *α*-diversity between donor samples and recipient mice revealed that mice receiving microbiota from patients with elevated C-reactive protein (CRP >  5  mg/L) had significantly lower *α*-diversity (Figure 4a). However, no significant difference was observed based on clinical activity (mild versus moderate-to-severe partial Mayo scores, Figure 4b). In contrast, *β* diversity analysis using Bray–Curtis distances revealed a small but significant difference between recipient mice from both patients with an elevated CRP level and those with a moderate-to-severe partial Mayo score (Mayo-score: *R*^2^ = 0.10, *p* < 0.0001; CRP: *R*^2^ = 0.08, *p* < 0.0001, Figure 4c, d). No significant associations between elevated CRP levels or moderate-to-severe partial Mayo scores and individual bacterial genera were found in the recipient mice 3 weeks after transfer.

Next, we investigated the relationships between bacterial taxonomical features and the inflammatory traits of the mice. To pinpoint bacteria potentially responsible for the colonic inflammation observed only in G8, we focused on species with significantly higher relative abundances in this group. Among the 18 bacterial features identified, five were more abundant in all the G8 mice than in all the other mice; these are specifically annotated as *Clostridioides difficile, Negativibacillus massiliensis, Bifidobacterium pseudolongum,* and two unclassified species belonging to the genera *Faecalitalea* and *Clostridium sensu stricto 1*. Given the known pathogenicity of *C. difficile*, we hypothesized that its clinically unrecognized presence in the G8 donor led to colonization of the recipient mice post-transfer, triggering an inflammatory response in the colon (Figure 4e). We confirmed the pathogenicity of the *C. difficile* species by performing qPCR for the genes encoding toxin A (*tcdA*) and toxin B (*tcdB*) in the feces of donors and recipient mice. Indeed, all the mice in G8 tested positive for both *tcdA* and *tcdB*, as did the donors in G8. The genes encoding binary toxins *cdtA* and *cdtB*, which are associated with more severe infection, were not detected in any of the samples (Supplementary Table 4). Given that the inflammatory phenotype in G8 was likely driven by *C. difficile*, we excluded G8 from correlations between the physiological traits and microbial features such as taxonomy or metabolic modeling.

We then explored further associations between bacterial abundance and mouse physiological traits in the remaining groups. Notably, the seven groups differed mainly in weight change and EWAT proportion; thus, bacterial genera in G1–G7 were correlated with these specific traits. Here, we observed a significant negative correlation of normalized EWAT weight with the genera *Eggerthella* and *Hungatella*, while the strongest positive correlation was with *Holdemania* (Figure 4f). Compared to the original donor abundances, both *Eggerthella* and *Holdemania* had lower abundances in the recipient mice, while *Hungatella* generally increased. However, all the strains exhibited low within-group variability upon ICC, indicating consistent transfer (Supplementary Tables 5 and 6).

In summary, we observed a strong group-specific effect of the engrafted bacteria, with notable similarities between the bacterial profiles of the original donor samples and those of the recipient mice. Beyond the transmission of a subclinical *C. difficile* infection observed in one animal group, specific bacterial genera of the human donors exhibited significant correlations with physiological traits in the remaining mice, especially those linked to weight.

### Effect of fecal transfer on skin and lung microbiota

**Figure 5.**
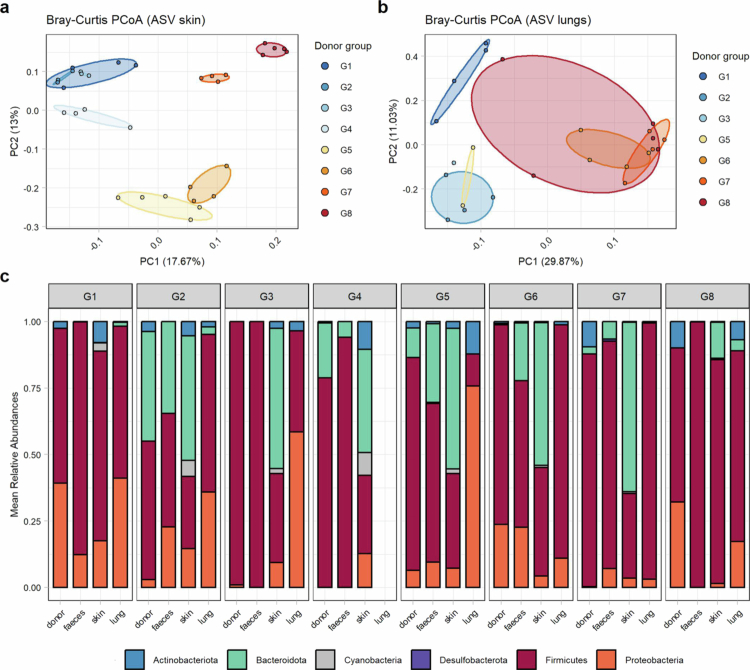
Bacterial engraftment after transfer to the skin and lungs. (a-b) Principal coordinate analysis (PCoA) based on the Bray‒Curtis distance of rarefied amplicon sequence variants (ASV) of (a) skin samples obtained from the ears (*n* = 32) and (b) from the lungs (*n* = 24) using 16S rRNA gene sequencing of the V1‒V2 region. (c) Mean relative bacterial abundance at the phylum level of the samples obtained per donor group of the donor stool and of the mouse feces, skin and lungs obtained three weeks after transfer using 16S rRNA gene sequencing of the V1‒V2 region.

We next analyzed bacterial community composition across the skin and lower airway of recipient mice to determine whether they clustered similarly to the fecal microbiome. Owing to low bacterial read counts, one sample from the skin and nine from the lung (all G4 samples) were excluded from the analysis. The Shannon index did not significantly differ in *α* diversity between donor groups in skin or lung tissue (*p* > 0.05, data not shown). However, *β*-diversity analysis revealed clear clustering between groups in the skin (R^2^ = 0.73, *p* < 0.0001) and to a lesser degree in the lower airways (R^2^ = 0.48, *p* < 0.0001, Figure 5a-b). Firmicutes were present at high relative abundances in all sample types, while Proteobacteria were generally present at a relatively high relative abundance in the lungs of the mice and a relatively low relative abundance in the mouse feces. Interestingly, Bacteroidota had preferential engraftment in the skin of recipient mice, with higher relative abundances than those found in human stool samples (Figure 5c). Notably, low read counts of the contaminant genus *Paenibacillus,* which were identified in the fecal samples of the mice prior to gavage, were found in the skin samples of most of the mice in groups 7 and 8 three weeks after microbial transfer. As only post-FMT fecal samples were additionally sequenced in the V1/V2 region, where contamination was not detected, the exact contaminant ASV could not be confirmed in the V1/V2 data of the skin samples. Nevertheless, as the donors of these two groups did not have identifiable *Paenibacillus* species, an isolated persistent low-level colonization on the skin seems likely.

### Physiological effects of FMT from Recipient Mice Stool

**Figure 6.**
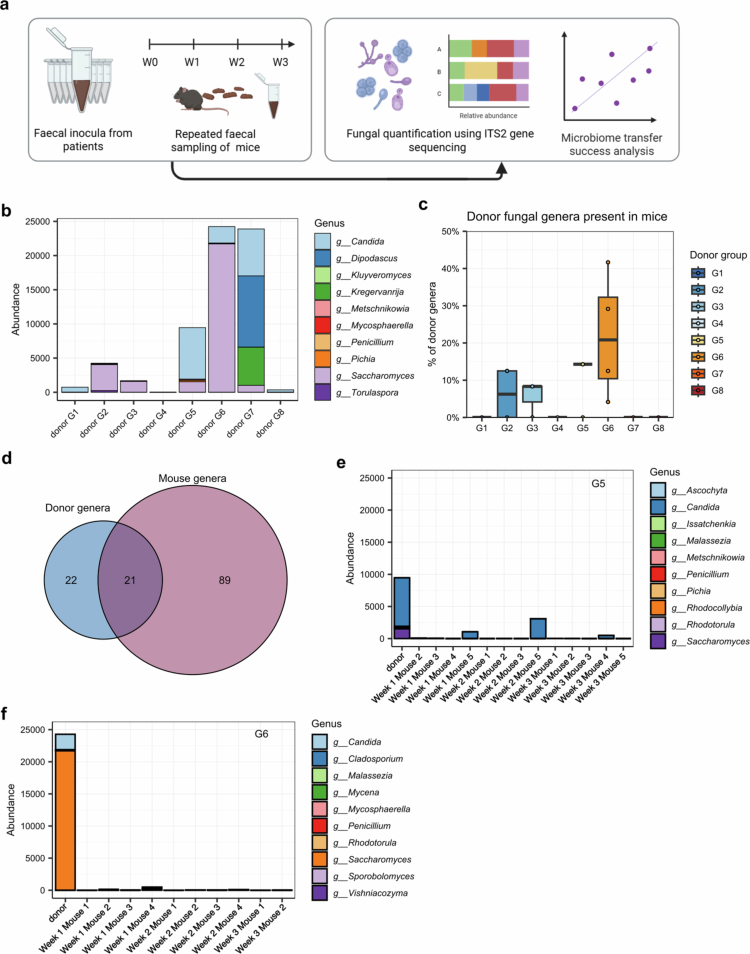
Fungal transfer analyses. (a) ITS2 sequencing of the fecal samples of the human donors and the weekly samples from the recipient mice was performed. (b) Absolute abundances of the ten most abundant fungal genera in the donor samples. (c) Proportion of fungal genera sequenced in the human samples that were successfully transplanted into at least one respective recipient mouse per donor group. (d) Total number of fungal genera sequenced in any mouse sample, human sample or both. (e, f) Absolute abundances of the ten most abundant fungal genera in group 5 (e) and group 6 (f) in the donors and their respective recipient mice longitudinally.

Our next goal was to characterize the fecal fungal communities in the human donors and recipient mice. We performed ITS2 gene sequencing on human samples and mouse samples from week 1, week 2 and week 3 after fecal transfer (Figure 6a). Given the high variability in absolute abundances and the generally low read counts in recipient mice, rarefaction was not performed after removing potential contaminants using the decontam R package. The absolute counts ranged from 2 to over 24,000 in the human stool samples and from 0 to 3,096 in the mouse fecal samples. The most abundant fungal genera in the donors were *Saccharomyces*, which was present in five donors, and *Candida*, which was found at varying abundances in all the donor samples (Figure 6b). The percentage of fungal genera transferred into recipient mice at any level clearly differed among the donor groups, ranging from 0% to 42% (Figure 6c). Among all the genera sequenced in the human and mouse samples, 21 were detected in both (Figure 6d). Analysis across all three sequenced timepoints revealed that successful fungal engraftment occurred in only a few individual mice (Figure 6e-f, Supplementary Figure 6). Owing to the generally low number of fungal reads in the recipient mice following FMT, the results were not subjected to further correlation analyses with mouse physiological traits and were omitted from subsequent analyses. Overall, the fungal community in recipient mice differed from that in the original human samples and was largely absent, indicating unsuccessful transmission.

### Metabolic modeling of bacterial communities

**Figure 7.**
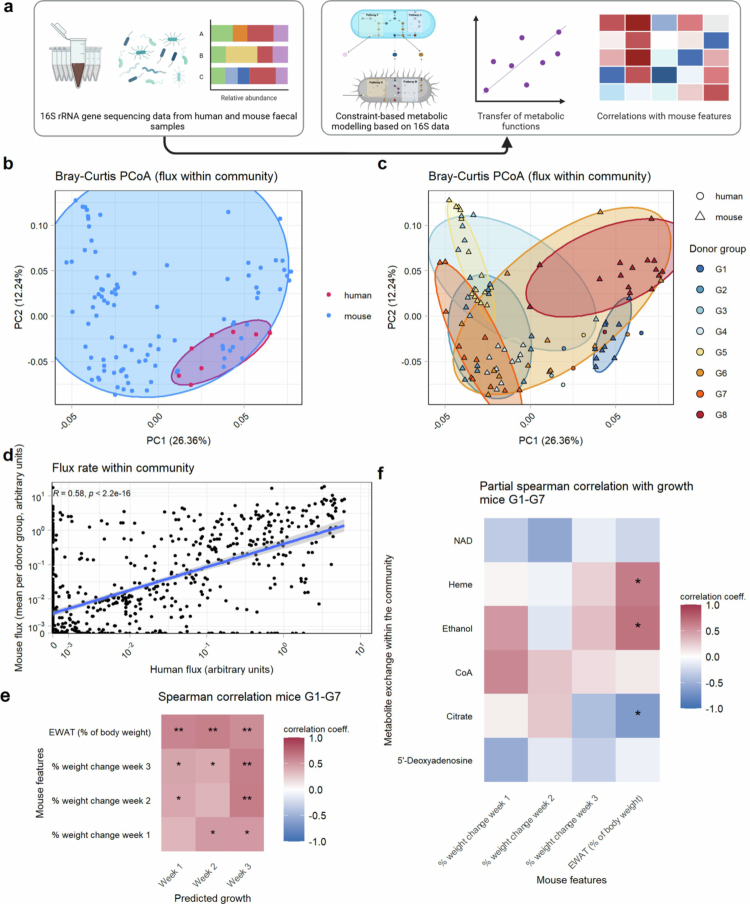
Constraint-based metabolic models of bacterial communities in the human donors and recipient mouse groups. (a) Taxonomic assignment and abundances based on 16S rRNA sequencing of the stool samples from the eight donors and weekly samples from 33 recipient mice were used to apply constraint-based metabolic modeling. One sample from G1 at week 2 was excluded because the predicted growth in the bacterial community was too low. (b, c) Principal coordinate analysis (PCoA) based on Bray‒Curtis distances of the predicted metabolic fluxes exchanged within the bacterial community of human and mouse stool samples, coloring by sample type (human or mouse) (b) or by donor group (c). (d) Scatterplot of all individual predicted metabolic fluxes of human samples and the mean abundances of their respective recipient mice after 3 weeks, Spearman correlation. One datapoint indicates one metabolic flux per human donor sample and the mean of their respective recipient mice. The blue line depicts the linear fit, and the gray shading represents the ±  standard error. (e) Spearman correlation of the physiological features and the predicted community growth in the murine stool samples of groups 1 to 7, followed by Benjamini‒Hochberg correction. (f) Partial Spearman correlation using mouse physiological features, predicted growth and predicted individual fluxes within the bacterial community of groups 1 to 7. (*:*p* < 0.05; **:*p* < 0.01; ***:*p* < 0.001). EWAT = epidydimal white adipose tissue.

Next, we wanted to characterize the microbial community at the functional level by analyzing the metabolic potential of the fecal bacteria, focusing on predicted metabolic fluxes and bacterial growth dynamics. To evaluate whether the predicted metabolic potential of the UC donor microbiota was retained in recipient mice, we analyzed the fecal bacterial communities from both donor samples and recipient mice via constraint-based metabolic modeling. The 16S rRNA sequencing data from the stool samples of the eight donors and the 33 recipient mice were used to predict the metabolic fluxes of the fecal bacteria exchanged within the community (Figure 7a). To examine differences between the predicted metabolic exchange in the bacterial community of the original donor samples and that in the recipient samples, the Bray‒Curtis distances of the fluxes within the bacterial community were computed. While there was an overlap of donor and mouse samples, the human samples clustered more closely together (*p* = 0.001) and differed in their distribution and centroid compared to the mouse samples (*R*^2^ = 0.04, *p* = 0.0011, Figure 7b). Analysis of mouse samples at the donor group level revealed a broader distribution within groups, with no significant variation across time points (*p* > 0.05). Additionally, donor groups explained less variance compared to the PCoA results for the 16S rRNA gene data (*R*^2^ = 0.51, *p* < 0.0001, Figure 7c). More importantly, the recipient mouse samples did not cluster as closely with their donor samples as the 16S rRNA gene data did (Figure 7c). Indeed, the donor sample of G3 even showed a significantly greater Bray‒Curtis distance to its recipient mice than to the mice from the other groups in terms of metabolic exchange in the bacterial community (Supplementary Figure 7, *p* < 0.0001). Nevertheless, when we compared the values of the predicted metabolic fluxes of the human samples and the mean flux heights of their respective recipient mice at week 3, we observed a significant positive correlation (Figure 7d). We correlated individual metabolic fluxes in mice with donor inflammation indices (elevated CRP/pMayo) and BMI adjusted for the donor group. However, no significant associations remained after BH correction (*p* > 0.05). Next, the predicted metabolic potential of the bacterial community as a whole was calculated in terms of growth, as defined by the potential maximal production of the community biomass per stool sample. We aimed to analyze whether the metabolic potential of the bacterial community, expressed as growth, influenced host physiology by correlating it with the weekly weight change of the mice and the relative EWAT weight of the mice in G1-G7, excluding G8 as an outlier considering the colitis phenotype. Here, we observed a uniformly positive relationship between the predicted growth and the mouse weight and EWAT (Figure 7e). When the individual predicted fluxes in the community were correlated with these mouse features, we found a significantly positive correlation between the relative EWAT weight and heme and ethanol metabolism in the respective communities (Figure 7f). Taken together, while constraint-based individual metabolic models from 16S rRNA gene data showed a lower correlation of donor and mouse features compared to the taxonomic level did, higher predicted metabolic fluxes correlated with mouse weight and EWAT mass.

### Variation in serum metabolic signatures of human donors and recipient mice

**Figure 8.**
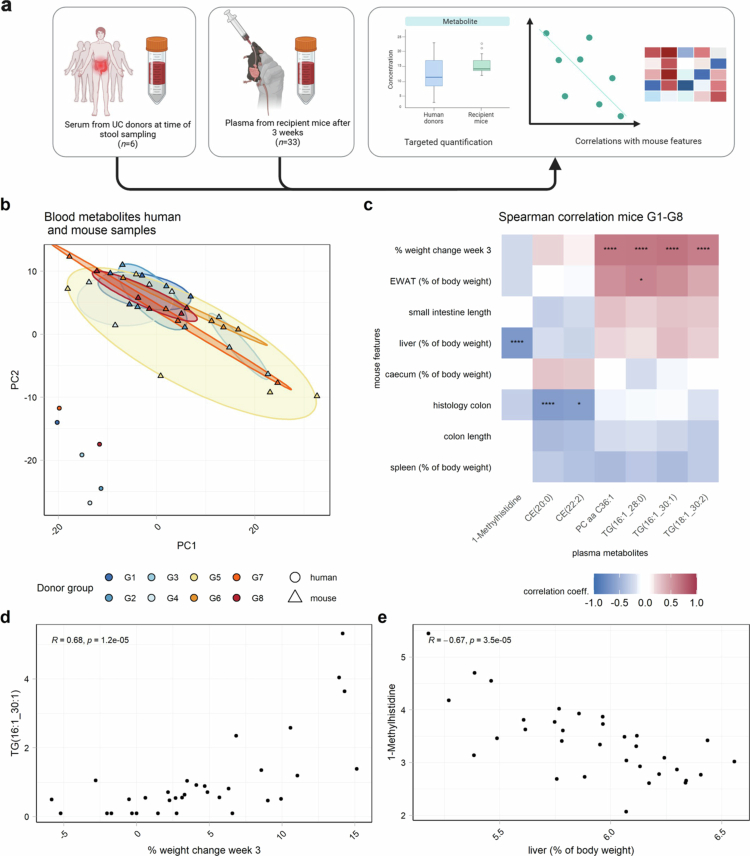
Blood metabolic signatures of human donors and recipient mice. (a) Visual representation of the experimental set-up. The metabolomics of the serum from six of the eight human donors and the plasma from the 33 recipient mice was measured in a targeted manner. (b) Principal component analysis (PCA) visualizing the differences between species and between donor groups in the mice. (c) Spearman correlation of the physiological features and plasma metabolites of the mice in groups 1 to 8 three weeks after microbiota transfer (*: *p* < 0.05; **: *p* < 0.01; ***: *p* < 0.001; ****: *p* < 0.0001). (d, e) Representative scatterplots showing specific significant correlations between the metabolite and mouse physiological features. TG = triglyceride; PC = phosphatidylcholine; CE = ceramide; EWAT = epidydimal white adipose tissue.

We next conducted targeted metabolomics analysis on plasma samples from all recipient mice that survived three weeks post-microbiota transfer (*n* = 33). In addition, six human donor serum samples were analysed, while donors G5 and G6 had to be excluded because they did not meet the quality control standards (Figure 8a). The aim was to assess similarities between human samples and their corresponding recipient mice as well as differences among recipient mice according to donor group. Therefore, we also included G8 in the analysis. Principal component analysis (PCA) showed a significant difference between human and mouse samples based on the centre-scaled metabolomics data (Figure 8b, R^2^ = 0.23, *p* < 0.001). However, only a marginal, nonsignificant signal was observed at a global scale between the mouse groups (Supplementary Figure 8, R^2^ = 0.28, *p* = 0.068). In addition, we did not find any significant associations of plasma metabolites in the mice with donor inflammatory features or BMI (BH-corrected *p* > 0.05). At week 3, analysis across all the mice, independent of group, revealed significant positive correlations between the percentage body weight change and multiple triglycerides, along with a phosphatidylcholine feature. This finding suggested that individual mouse physiology had a stronger influence on metabolite profiles than did donor group membership (Figure 8c-e).

### Integrative variance partitioning analysis identifies group-specific individual metabolic traits linked to bacterial colonization events

**Figure 9.**
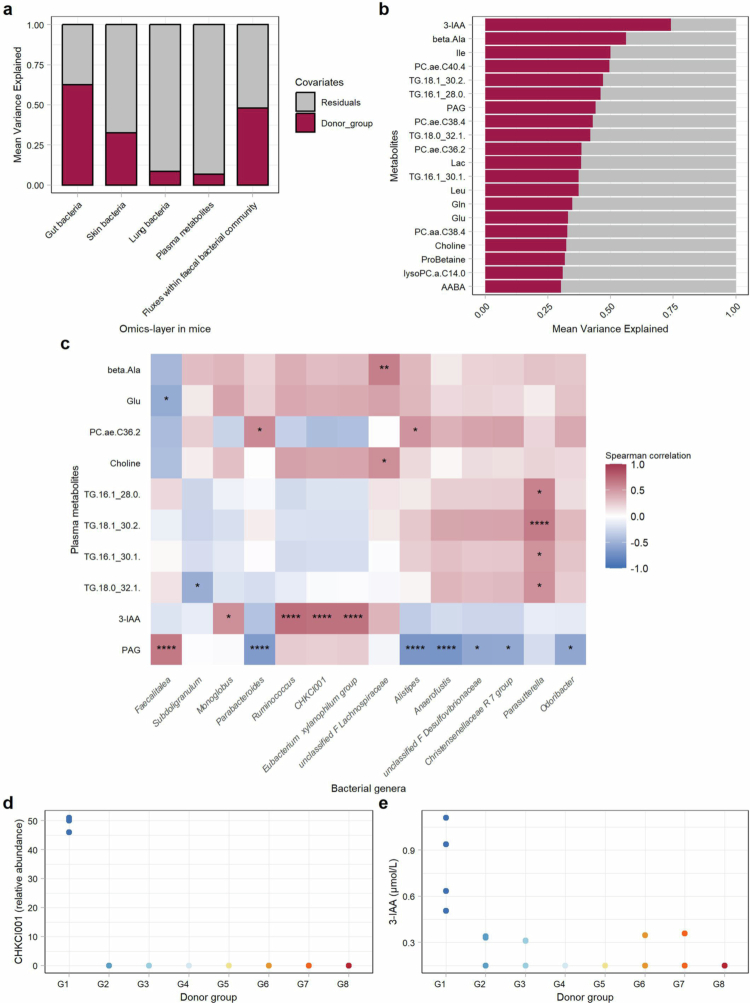
Variance partition analysis (VPA) of the donor group effect. (a) VPA of the recipient mice at various omics layers to assess the variance explained by the donor group three weeks after fecal transfer. (b) The top 20 plasma metabolites with the greatest variation associated with the donor group identified in the VPA. (c) Spearman correlation between the top 20 most variable metabolites and bacterial genera in the feces per donor group, followed by Benjamini‒Hochberg correction. Only significantly correlated genera or metabolites are shown (*:*p* < 0.05; **:*p* < 0.01; ***:*p* < 0.001; ****:*p* < 0.0001). (d, e) Representative visualization of the genus *CHKCI001* in the donor group and the strongly positively correlated metabolite 3-indoleacetic acid (3-IAA).

To systematically assess the impact of individual donor microbiota, we conducted an integrative variance partition analysis (VPA) on all eight mouse groups. Over 60% of the variation in the fecal bacteria at the genus level was explained by the donor group. This proportion decreased to 33% in the bacteria sequenced in the skin, while 9% of the variation was explained by the donor group in the lungs of the mice. The metabolic modeling revealed a substantial donor group effect, accounting for 48% of the variance in the predicted metabolic fluxes within the community. Plasma metabolomics showed the weakest group effect of all the multiomics layers, explaining only 7% of the variance. Figure 9a).

To further investigate the subtle group-specific effects, we extracted the 20 plasma metabolites that accounted for the highest variation explained by the donor group. We observed that individual metabolites explained a high proportion of the variance explained by the donor group, with up to nearly 75% of the variance explained (Figure 9b). To further characterize the effects of the bacterial communities on the plasma metabolite profiles of the mice, we correlated these 20 metabolites with the 20 most variable fecal bacterial genera as determined by VPA (Figure 9c). We identified a distinct group-related pattern in which the isolated increase in one genus in one mouse group was mirrored by a clear increase in the respective correlated metabolite in that same mouse group. Examples include the genera *CHKCI001* and *Ruminococcus* and the metabolite 3-indoleacetic acid (Figure 9d-e). Furthermore, *Parasutterella* was significantly positively correlated with several triglycerides in the plasma. Interestingly, *Parasutterella* was more abundant in the recipient mice compared to the donor mice and presented low intragroup variation and high between-group variation in the ICCs (Supplementary Tables 5 and 6). Taken together, although the overall effect of the donor group on plasma metabolomics was relatively weak, distinct bacterial genera specific to each group were clearly associated with corresponding group-specific metabolites. These associations suggest that even subtle microbial differences may influence metabolite profiles and, in turn, contribute to the physiological heterogeneity observed among recipient mice.

## Discussion

Fecal microbiota transfer studies from humans to mice serve as crucial tools for investigating perturbed host‒microbiota interactions in IBD and their physiological consequences *in vivo* under well-controlled conditions. However, these studies are inherently heterogeneous and difficult to replicate. Systematic longitudinal studies on colonization and direct comparisons of donor microbiomes with recipient communities and their phenotypic consequences in mice are largely lacking, making it difficult to evaluate the fidelity and stability of cross-species microbial engraftment. Addressing this gap is essential for improving the reproducibility and translatability of microbial transfer research. In this study, we employed a structured multiomics approach, including 16S rRNA gene and ITS2 amplicon sequencing alongside targeted plasma metabolomics, to investigate microbial and metabolic profiles in mice inoculated with fecal microbiota from eight UC donors with varying disease activity.

Except for a single experimental group, no signs of intestinal inflammation were observed during the colonization period, indicating that microbial engraftment alone was largely insufficient to induce inflammation. In the only group showing signs of inflammation, we found a subclinical *C. difficile* infection of the donor as the likely explanation for the outlier phenotype. The group with the incidental *C. difficile* infection consisted of only five mice, we found consistent colonization in all the mice. Mouse models have been used to study *C. difficile* infections, though the induction of inflammation usually requires antibiotic administration when mice have a normal murine microbiome or are colonized with a healthy humanized microbiome.^69^^,^^70^ Although based on a small experimental group, our findings on the tendency of *C. difficile* to spontaneously expand and potentially trigger a pathogenic response are consistent with a recent report in mice transplanted with fecal samples from CD patients.^24^ This highlights the need to distinguish the transmission of *C. difficile* or other opportunistic pathogens from broader microbiota effects when evaluating the impact of microbial transfer in IBD patients.

Assessing the transfer success and dynamics of colonization by 16S rRNA amplicon sequencing, we show reduced *α* diversity in recipient mice compared to human donors, with 70.8% of bacterial genera from donors detected in at least one mouse. This result is consistent with a previously published meta-analysis that reported that the average transfer success rate at the genus level was approximately 60% in GF mice.^71^ Administration of the fecal inocula via gavage was selected according to common practices and to avoid the risk of mucosal injury through rectal administration.^72^^‐^^74^ Mice receiving microbiota from donors with elevated CRP levels presented significantly lower Shannon diversity than did those from donors with normal CRP levels. This trend, already present in donor samples, was maintained and amplified in recipients, mirroring UC patients, where higher inflammation is correlated with reduced microbial diversity.^75^

*β*-Diversity analyses revealed strong within-group similarities among recipient mice and close proximity to their respective human donors, indicating at least partial retention of individual taxonomic profiles. Longitudinal analysis showed minimal impact of sampling time, suggesting rapid colonization with stable microbial patterns emerging as early as the first week. *Hungatella* presented the highest relative abundance in mice, while *Prevotella* spp. and *Faecalibacterium* were less abundant, partially recapitulating the meta-analysis by Li.^71^ Intraclass correlation (ICC) analysis revealed distinct patterns of engraftment variability, e.g., *Bacteroides* presented high inter-group variability but low intra-group variability, with successful transfer linked to its donor abundance. This finding is in line with the results of a meta-analysis by Ianiro et al., who reported that members of the *Bacteroides* genus are top-engraftment species in heterologous FMT for a variety of conditions.^76^ In contrast, *Turicibacter* presented high within-group variability and significant stochasticity in its transfer, reflecting incomplete and highly variable engraftment. These results underscore the complex dynamics and variability in microbial transfer and highlight the necessity of an adequate number of replicate mice per individual donor.

Interestingly, recipient mice exhibited diverse physiological trajectories following FMT. Variation in weight development among mice transplanted with human fecal microbiota has been previously reported and is often linked to differences in the BMI of the original donor. For example, stool from BMI-discordant twins induced a higher fat mass in recipient mice from obese twins than in those from lean twins.^77^ Notably, the weight trajectories of our recipient mice did not show associations with the human donor’s BMI, age or inflammation level. Individual mouse weight changes and relative EWAT masses were significantly associated with certain bacterial genera. First, a strong negative correlation with the genera *Eggerthella, Hungatella* and *Morganella* was detected. This finding reflects the findings of a meta-analysis in which *Eggerthella* was shown to be significantly less abundant in obese individuals.^78^ *Hungatella* was shown to be inversely correlated with body weight in a mouse model of obesity and breast cancer.^79^ One study indicated a positive correlation of *Morganella* with weight in mice receiving several synthetic bacterial isolates from obese individuals.^80^ The genera *Holdemania* and *Faecalitalea*, on the other hand, were positively correlated with weight in the recipient mice in our study. *Holdemania* was one of the genera preserved in FMT from obese patients into GF mice in a study in which the mice gained weight after FMT.^81^ The genus *Faecalitalea* was found to be more abundant in obese humans in a large meta-analysis.^82^ Notably, the associations between complex clinical donor phenotypes (including weight), medications, microbiota and physiological responses in colonized mice highlight the need for future larger studies with more deeply phenotyped donors, higher numbers of recipient mice and, ultimately, the causal effects of single isolated strains. Given our study set-up, overinterpretation of the observed clinical–microbiota associations should be avoided.

We also examined bacterial engraftment beyond the gut by collecting skin and lung samples three weeks after FMT. While group clustering was maintained in both tissues, microbiome composition significantly differed from that of the gut, reflecting the well-known variability across body sites shaped by unique physiological conditions.^83^^,^^84^ In the lungs, we observed high inter-group heterogeneity, with the dominant phyla Firmicutes and Proteobacteria aligning with findings of lung microbiome composition in mice. ^85^ In the skin, the most abundant phyla were Proteobacteria, Firmicutes and Bacteroidota. Although present in the original donor feces, we could not detect Actinobacteria at high relative abundances in the mouse skin, unlike our previous study on native murine skin microbiota, in which both wild-type mice and laboratory mice had a high proportion of Actinobacteria.^40^ Notably, Actinobacteria were virtually absent in mouse feces as a potential source for secondary skin colonization, likely because bacterial taxa are originally adapted to the human gut environment, which could contribute to this difference in native mouse skin microbiota. Our findings highlight the reliance of the microbiota at other body sites on gut inoculation, which may influence immune phenotypes in gnotobiotic studies. This underscores the need for colonization strategies beyond oral gavage to better mimic the physiological microbiota distribution.

ITS2 sequencing of fecal fungal communities revealed a heterogeneous read count across donor samples, confirming the low biomass nature of the human gut mycobiota. Some donor samples even yielded insufficient read counts, which is consistent with findings from previous studies.^86^ The fungal genera with the highest relative abundances in the donors reflected those described in the general population, such as *Candida* and *Saccharomyces*.^86^ Studies analysing the transfer of such fungal components from the human microbiome into GF-mice are scarce. We observed either low or no reads in the recipient mice, which is in line with other results. *Saccharomyces cerevisiae* found in human CD donor samples did not engraft in recipient GF-mice.^24^ Another study using stool from neonates with bronchopulmonary dysplasia also found wide heterogeneity in fungal read counts from recipient mouse fecal samples.^87^ In a cotransfer experiment with a synthetic community of 12 bacterial and 5 fungal species, the fungal populations declined significantly over time, ultimately reaching levels tenfold lower than the bacterial populations after 9–10 weeks.^88^ Notably, as in most studies, DNA extraction in our work was optimized for bacterial DNA, and a larger amount of fecal material may be necessary to capture the low-biomass fungal community sufficiently. Although no conclusions can be drawn regarding the exact functional role of the transplanted mycobiome, the incomplete transfer of fungi into GF mice should clearly be taken into account when interpreting microbiome transfer experiments in IBD models.^89^^,^^90^

Metabolic modeling of flux exchanges within the bacterial community provides functional insights beyond the taxonomic data obtained from 16S rRNA gene sequencing. We previously showed that prediction of metabolic cooperativity in IBD patients via constraint-based modeling could be used to anticipate their response to anti-TNF treatment.^18^ Variance partition analysis in our set-up revealed a strong effect of the group on the predicted fluxes, indicating that alterations in the bacterial community in the mice follow a reproducible pattern when the same original human stool sample is transplanted. In our study, the predicted bacterial growth was strongly positively correlated with the weight change and relative EWAT weight of the recipient mice. These findings suggest that a higher metabolic productivity in the bacterial community benefits the host in terms of weight gain. This phenotype correlates with heme flux, a key driver of intestinal iron absorption, metabolism, oxygen transport, electron transfer, and antioxidant activity.^91^ However, our analyses also suggest that predicted bacterial metabolic functions exhibit even greater divergence from the donor community than do taxonomic differences. While metabolic modeling can only make *in silico* predictions based on the potential reactions in the bacterial community and cannot reflect actual metabolite concentrations, *in vitro* validation of gapseq has previously shown an accuracy of 81%.^49^ Our findings align with those of Nagao-Kitamoto et al., who used PiCRUSt to show that predicted metabolic differences in the human microbiota do not fully translate to their humanized gnotobiotic mice. This highlights a key limitation of studying host-microbe metabolism in gnotobiotic mice, as individual metabolic functions may be consistently over- or underrepresented despite successful bacterial taxon transfer.

At the blood metabolome level, the donor group had only a minimal impact on recipient plasma metabolite signatures. Differences in input material (human serum vs. mouse plasma) limit direct donor–recipient comparisons in addition to species-level metabolic differences. However, we found clear associations between individual metabolites and specific mouse traits. Several triglycerides were positively associated with weight gain, while two ceramides show a negative correlation with increased colonic inflammation scores. Ceramides are crucial for maintaining intestinal barrier function and immune modulation, and their alterations have been previously reported in IBD patients.^92^^,^^93^ Although the overall group effect and thus the potential global influence of the gut microbiota on the metabolic profile are minimal, we identified significant associations between the presence of specific plasma metabolites and highly variable bacterial taxa. Notably, 3-IAA, a tryptophan metabolite, showed a strong positive correlation with *Ruminococcus*, particularly *Ruminococcus gnavus*, which degrades tryptophan to tryptamine, a precursor of 3-IAA.^94^^,^^95^ Furthermore, a strong positive association with *Eubacterium xylanophilum* group was observed, which was previously negatively associated with fecal tryptophan.^96^ These findings are of particular interest, as 3-IAA levels have been found to be reduced in patients with IBD, and supplementation of the metabolite has shown immunomodulatory effects in mouse models of IBD.^97^^,^^98^ Another interesting finding was the positive correlation between the genus *Parasutterella* and several triglycerides. This mirrors findings from a cohort of obese human patients linking high *Parasutterella* abundance with activation of the human fatty acid biosynthesis pathway, suggesting a mechanism for carbohydrate-dependent body weight gain.^99^

While this study offers important findings, several limitations must be acknowledged to contextualize the results. Immediately prior to FMT, we detected bacteria from the genera *Bacillus* and *Paenibacillus* in four of the eight mouse groups, two known contaminants in germ-free facilities.^100^^‐^^103^ Importantly, we show that this contamination did not persist after gavage in the fecal microbiome. The mice were consciously included in the analysis, as they did not show systematic differences in terms of physiological characteristics or any of the reported traits. As the aim was not to study GF mice *per se*, we overtly point to this experimental limitation but still expect our results to be valid for our purpose, which is further supported by a number of studies using antibiotic-treated mice for fecal microbial transfer studies.^104^ Second, it is important to note that the metabolic modeling tool used to infer the functional potential of the fecal microbiota was primarily developed based on human-derived bacterial data and their physiological context. Because donor stools exhibited slightly higher diversity, a greater proportion of their bacteria mapped to the reference catalogue than did those from recipient mice. This may introduce bias in comparisons of metabolic potential between human and humanized murine microbiota. Although our models exclude assumptions about human host metabolism, are corrected for nutritional input and thus are conceptually appropriate, care is needed when these metabolic predictions are applied to murine physiology. Third, the case-only design limits direct comparison to studies where microbiota from healthy individuals are transferred, as our initial focus was on the potential transmission of activity-dependent microbial features in UC patients. Additionally, our results emphasize the need for discretion regarding nonbacterial components of the microbiome, such as fungi, which could play an important role in perturbed host–microbiota interactions, particularly in IBD.^89^^,^^90^ Finally, caution is needed when extrapolating our results from mouse studies to humans, including UC patients, as the physiological associations observed in mice may differ significantly owing to host species effects and the chronic nature of inflammation. The current experimental design with a follow-up of a few weeks is inadequate for assessing the long-term dynamics of microbial composition and their sustained effects on inflammation.

Our multiomics study significantly contributes to the understanding of the fidelity and stability of cross-species microbial engraftment upon human FMT into gnotobiotic mice. Using within- and between-group comparisons of longitudinal 16S rRNA gene datasets, we highlight bacterial taxa that display stable or highly variable colonization patterns. Combining taxonomic and metabolomic data, we show that the presence or absence of individual bacteria is associated with significant variation in plasma metabolites such as 3-IAA. Furthermore, we highlight the importance of analysing bacterial metabolic networks and engraftment beyond the gut. In conclusion, while FMT of human material into mice consistently transmits certain bacterial features, it also introduces alterations due to systematic biases during colonization and stochastic effects. These factors must be considered when interpreting findings from humanized gnotobiotic mice.

## Supplementary Material

Supplementary material Supplementary figures.

Supplementary material Supplementary_Tables11_Nov_2025_15_06

## Data Availability

All supplementary material has been uploaded to Figshare: DOI for supplementary tables: https://doi.org/10.6084/m9.figshare.29873510 DOI for histological images: https://doi.org/10.6084/m9.figshare.29890274 The sequencing data that support the findings of this study will be openly available in the Sequence Read Archive (SRA) with the following project number: PRJNA1248768. The following link provides reviewer access to the BioProject: https://dataview.ncbi.nlm.nih.gov/object/PRJNA1248768?reviewer=1vl9hg95nf37nrlt0jpdestv7j Further data used for analysis can be found in the following supplementary tables, available in the Figshare upload: https://doi.org/10.6084/m9.figshare.29873510 Supplementary Table 7: Rarefied ASV table of the 16S gene sequencing data (V3/V4) of faecal samples. Supplementary Table 8, 9, 10, 11: Rarefied ASV table of the 16S gene sequencing data (V1/V2) of donor samples, and mouse faecal, skin, and lung samples respectively. Supplementary Table 12: Unrarefied ASV table of ITS2 gene sequencing of donor and murine faecal samples. Supplementary Table 13: Metabolomics data of human serum and murine plasma after data cleaning and filtering as described in the methods. Supplementary Table 14: Metabolic modeling fluxes exchanged within the bacterial community in human and murine faecal samples and their respective predicted growth per sample. Supplementary Table 15: Conversion table for the metabolic modeling flux names. Supplementary Table 16: Data of the respective human donor per recipient mouse. Supplementary Table 17: Physiological data of the recipient mice and fecal lipocalin-2 concentration of the mice.
