# Supplementary material for: Cross-species engraftment biases and metabolic divergence in gnotobiotic mice humanized with ulcerative colitis microbiota
Source: Gut Microbes. 2025 Nov 24;17(1):2581445. doi: 10.1080/19490976.2025.2581445 (PMC12645861; doi:10.1080/19490976.2025.2581445)
Supplement: Supplementary material — Supplementary figures. [file KGMI_A_2581445_SM8422.docx]

**Supplementary figures**

Group 1


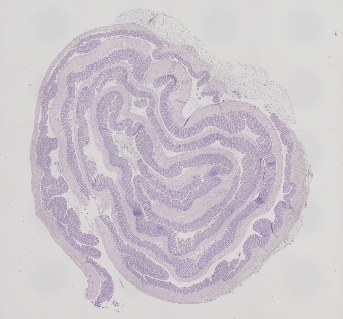

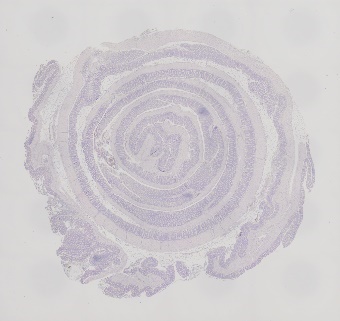

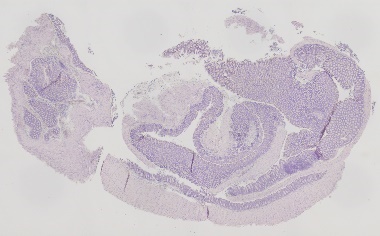

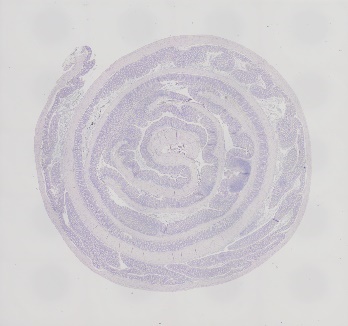


Group 2


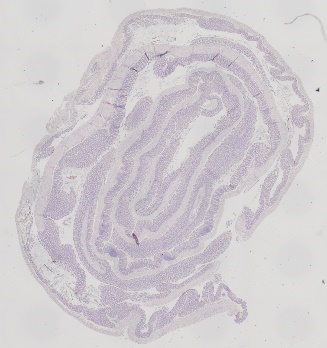

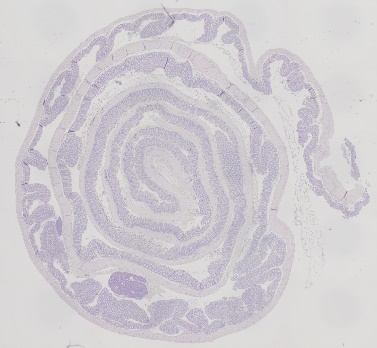

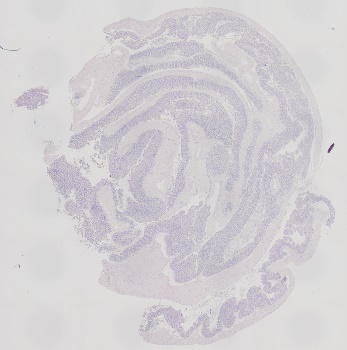

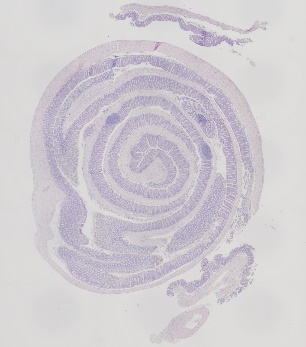


Group 3


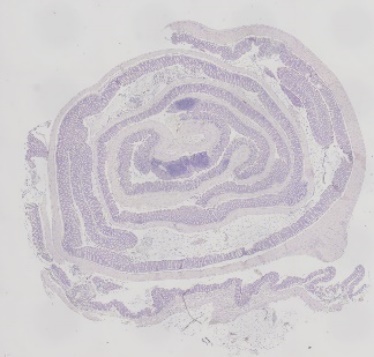

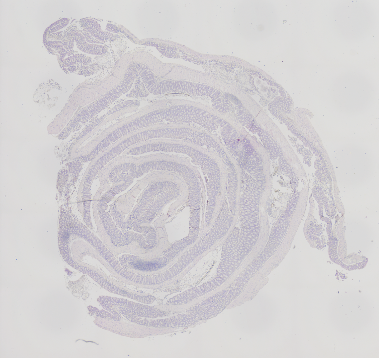

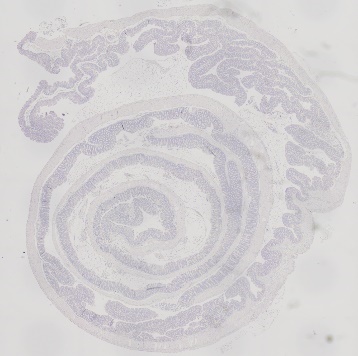


Group 4


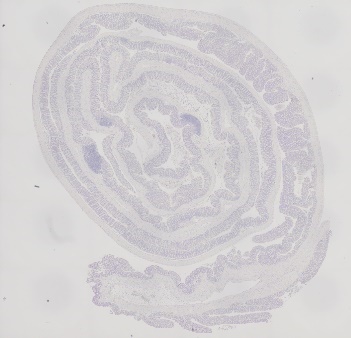

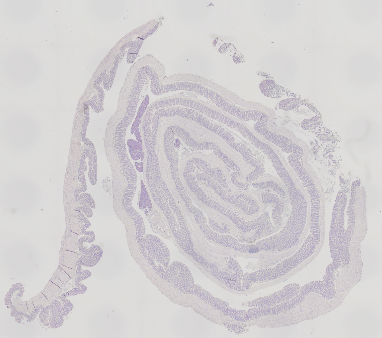

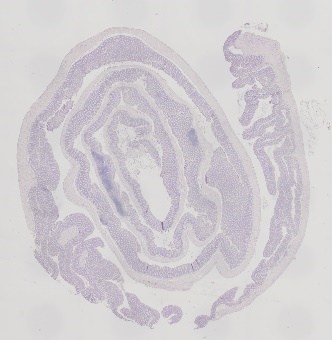

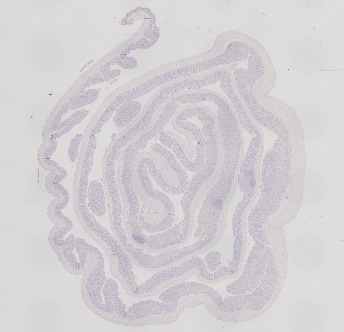


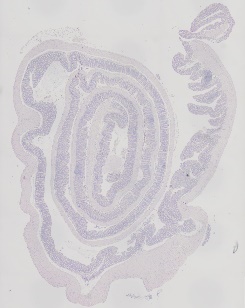

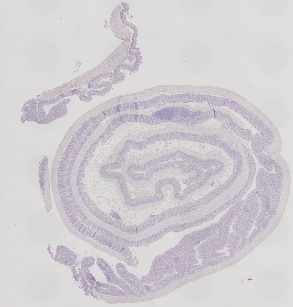

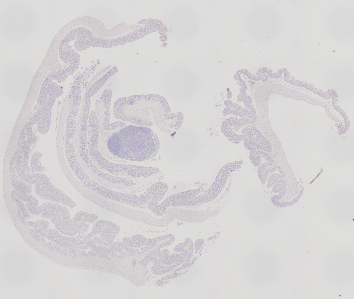

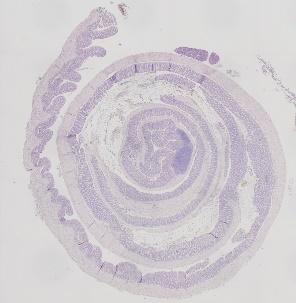

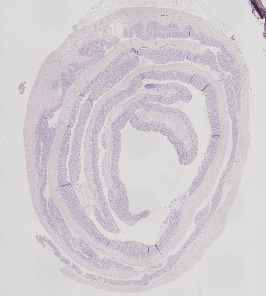


Group 6

Group 5


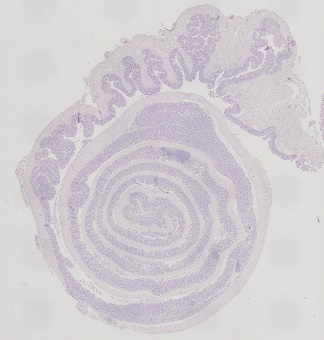

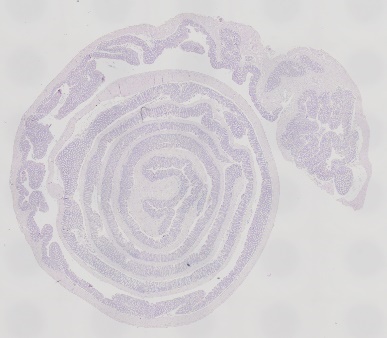

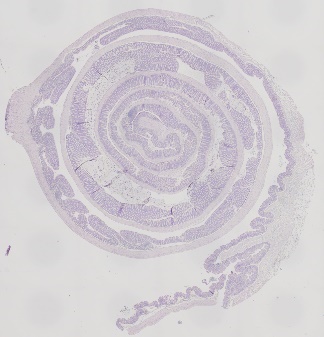

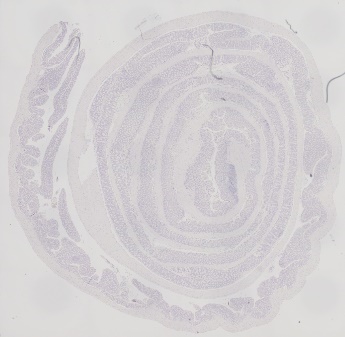


Group 7


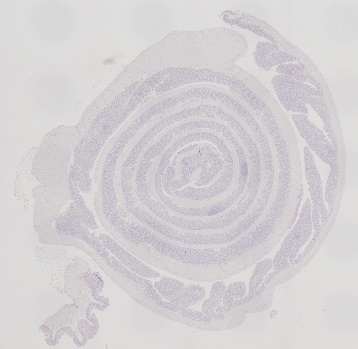

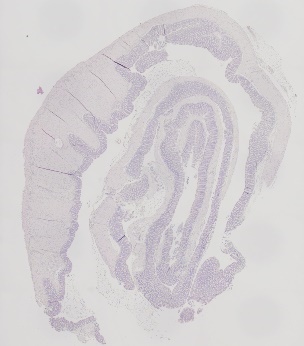

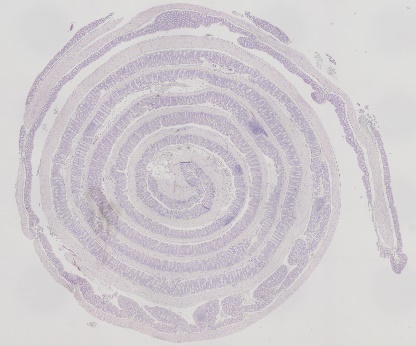

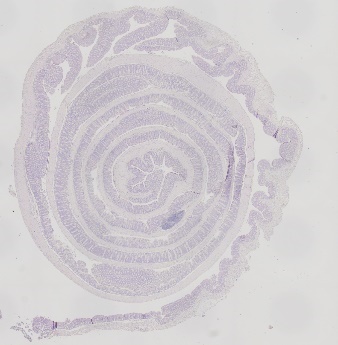


Group 8


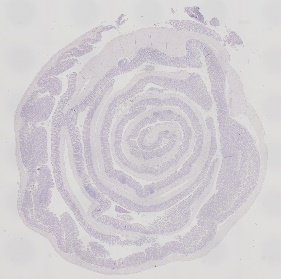

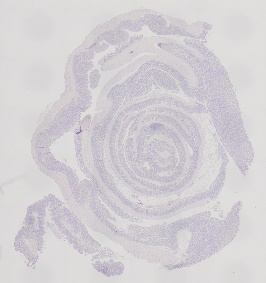

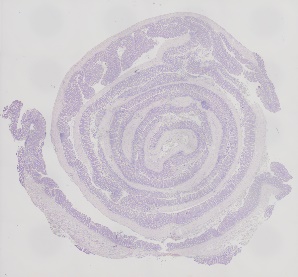

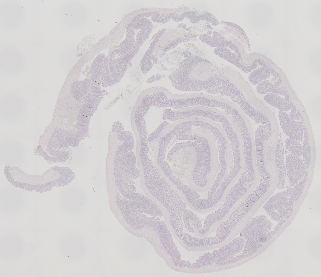

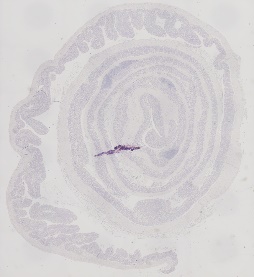


Supplementary Figure 1. H&E staining of colonic Swiss Rolls of recipient mice, one image per mouse in numerical order, sorted by donor groups.


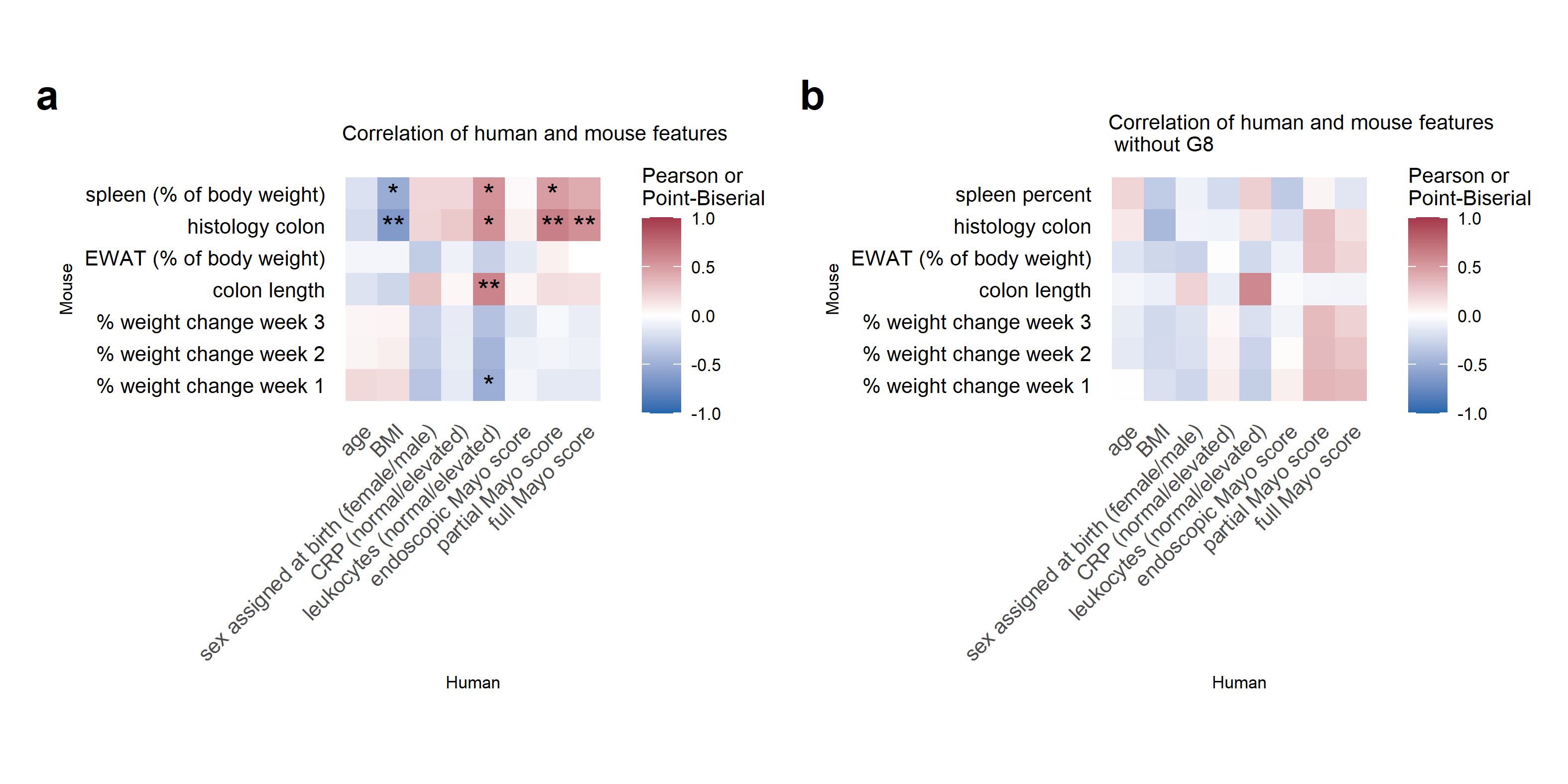


Supplementary Figure 2. Pearson correlation for continuous and point-biserial correlation for binary human features with the mouse physiological features that significantly differed between donor groups. (a) all mice, (b) excluding mice from donor group 8. (*: p<0.05; **:p<0.01; ***:p<0.001; ****:p<0.0001)


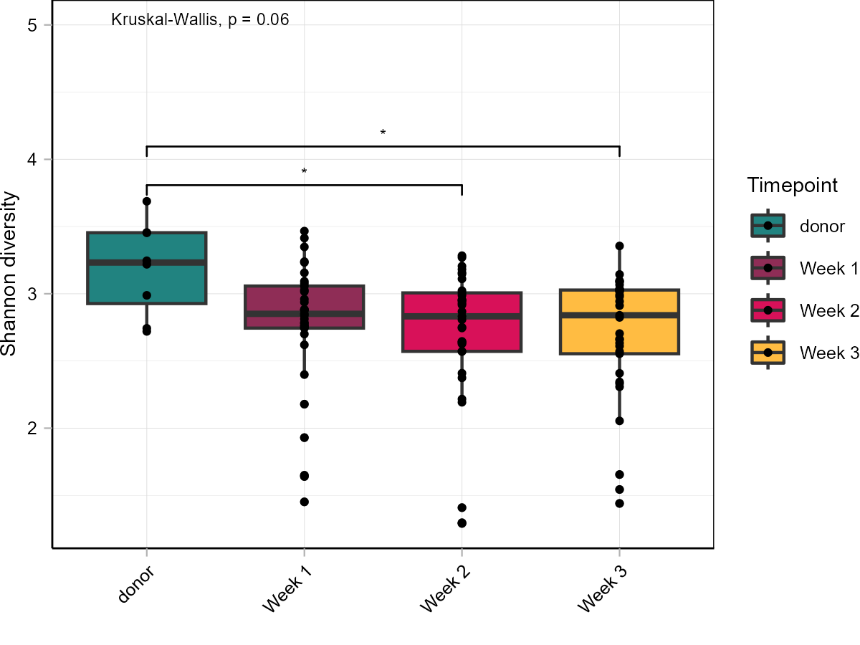


Supplementary Figure 3. Shannon diversity index grouped by donor samples and mouse samples at week 1,2, and 3 after faecal transplantation, Kruskal-Wallis test followed by Dunns’test. (*:p<0.05; **:p<0.01; ***:p<0.001; ****:p<0.0001)


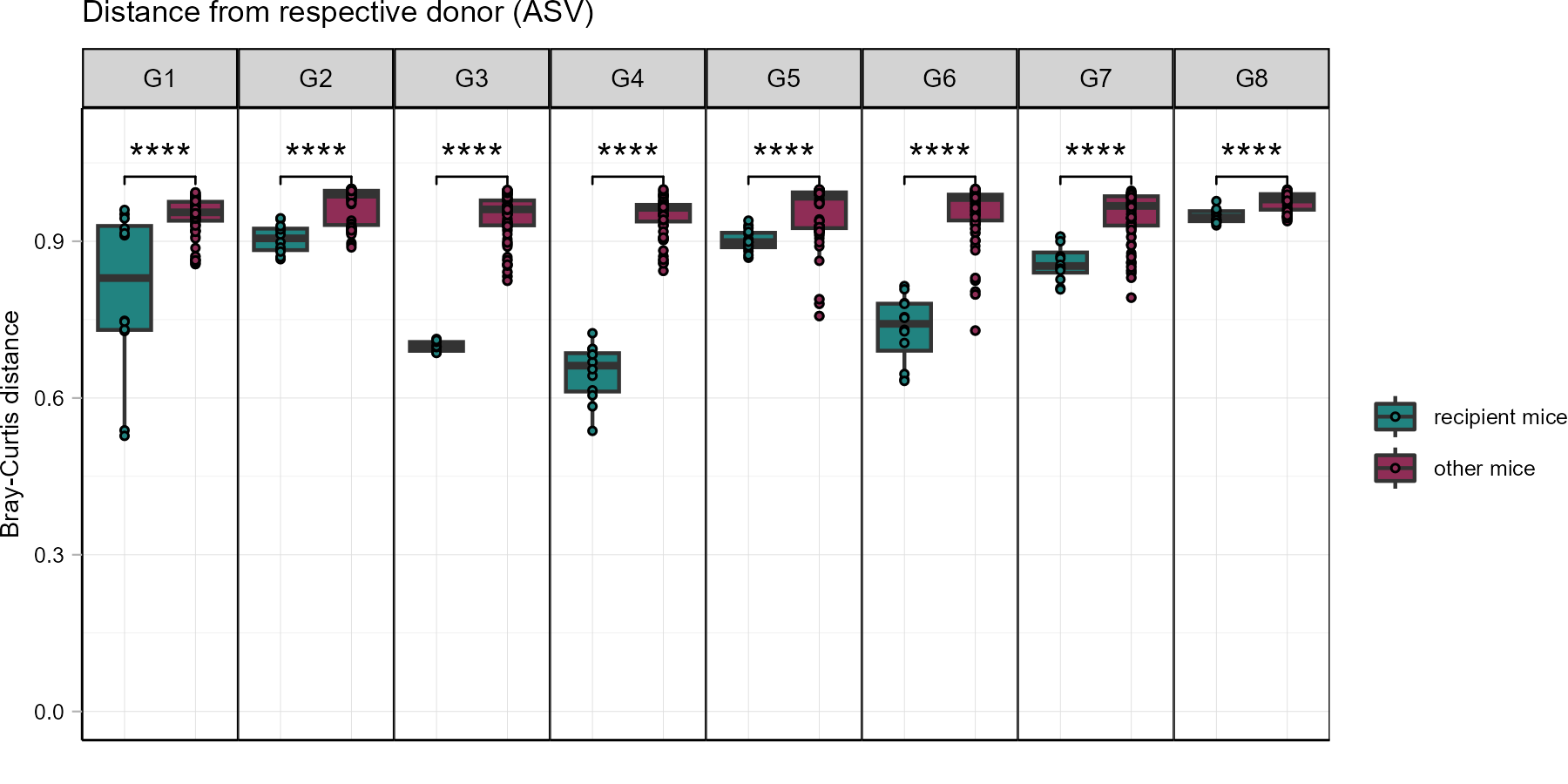


Supplementary Figure 4. Bray-Curtis distance of faecal bacterial ASVs from the donor sample to the respective recipient mice or to all other mice from the remaining seven donor groups, plotted per donor group, all sampling timepoints after gavage included. BH-corrected Wilcoxon test. (*:p<0.05; **:p<0.01; ***:p<0.001; ****:p<0.0001)


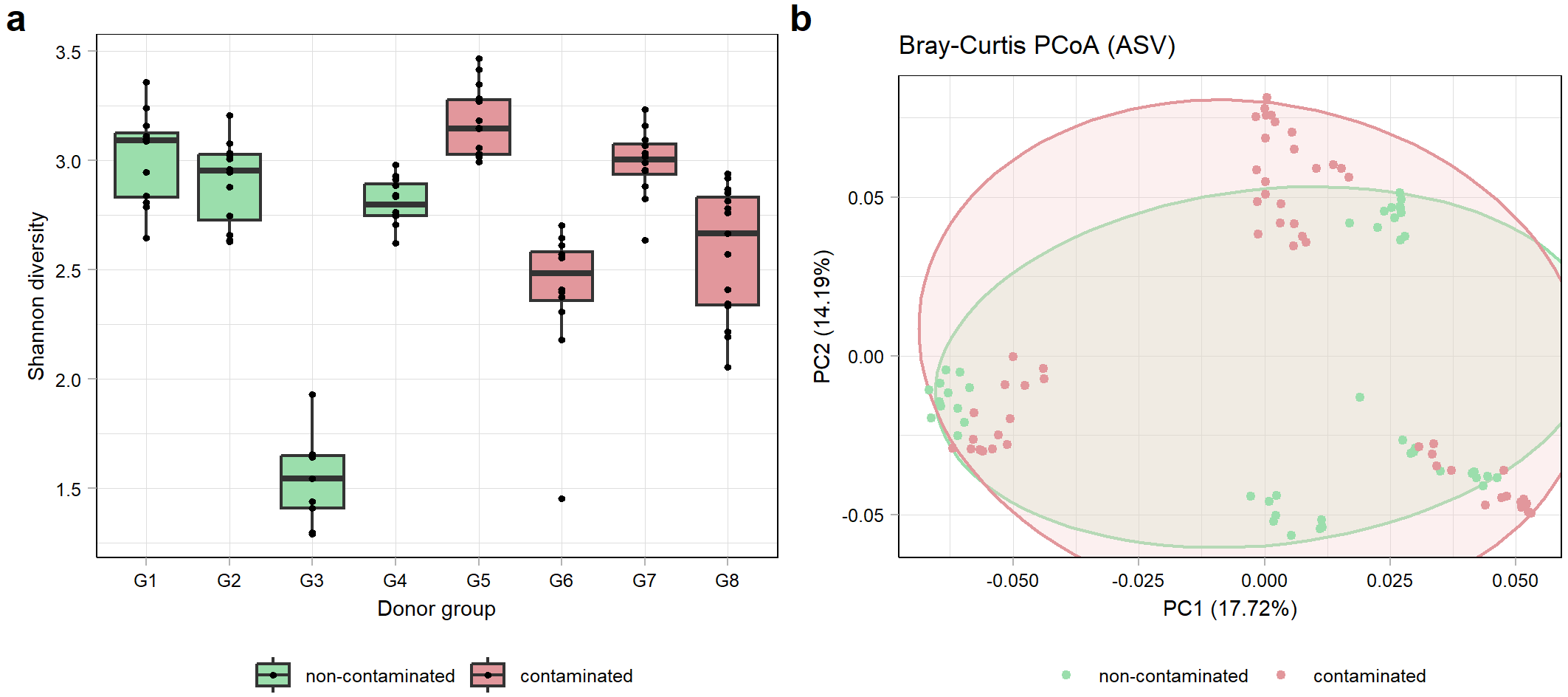


Supplementary Figure 5. (a) Shannon diversity of the mouse samples per group at all three sampling timepoints after gavage, colours indicating the contaminated and non-contaminated groups before gavage. (b) B-diversity of recipient mice after microbiome transfer at all three sampling timepoints after gavage.


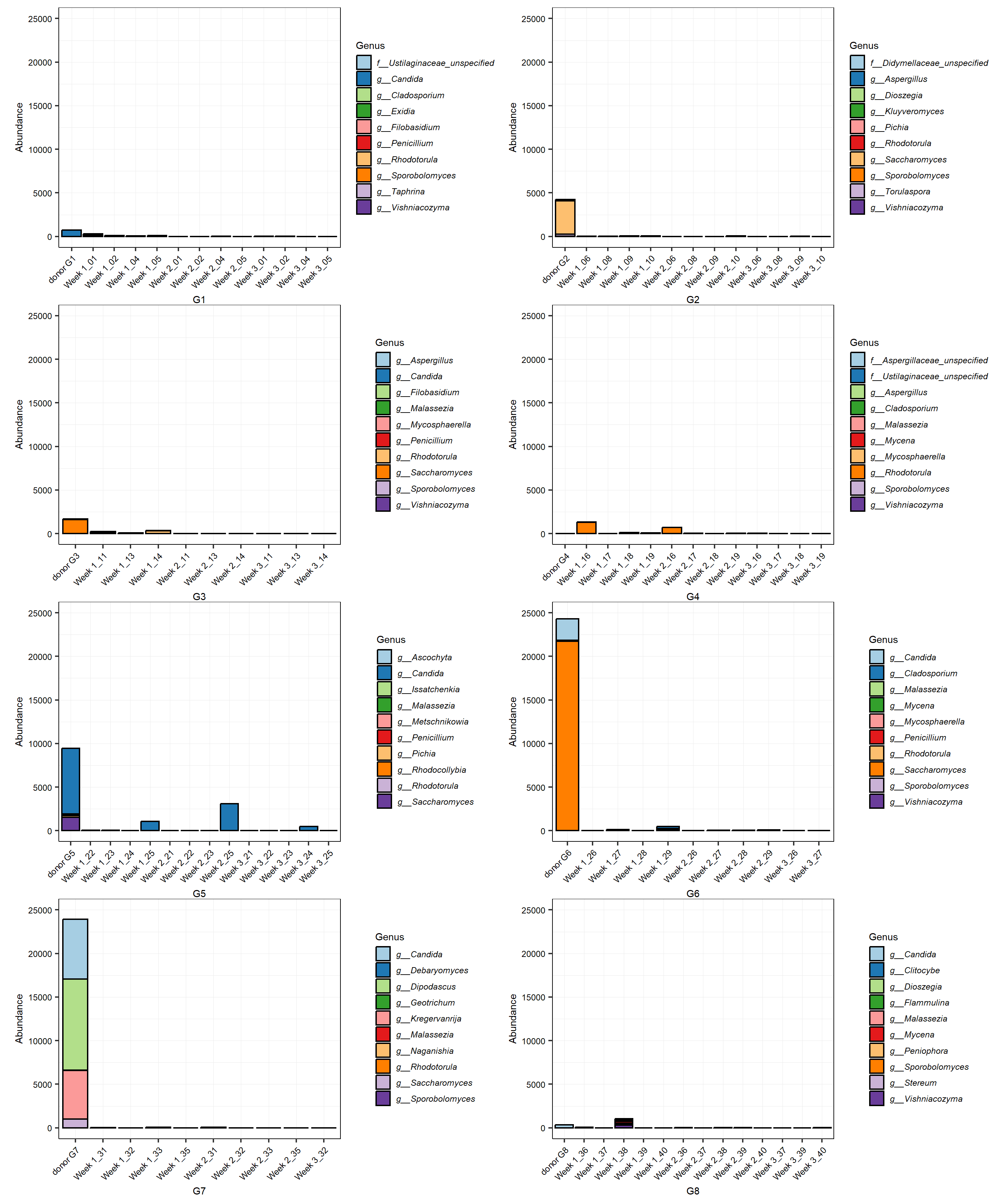


Supplementary Figure 6. Stacked barplots of unrarefied fungal genera sequenced upon ITS2 in donor sample and respective recipient mice per donor group, number after underscore represents an individual mouse in the donor groups.


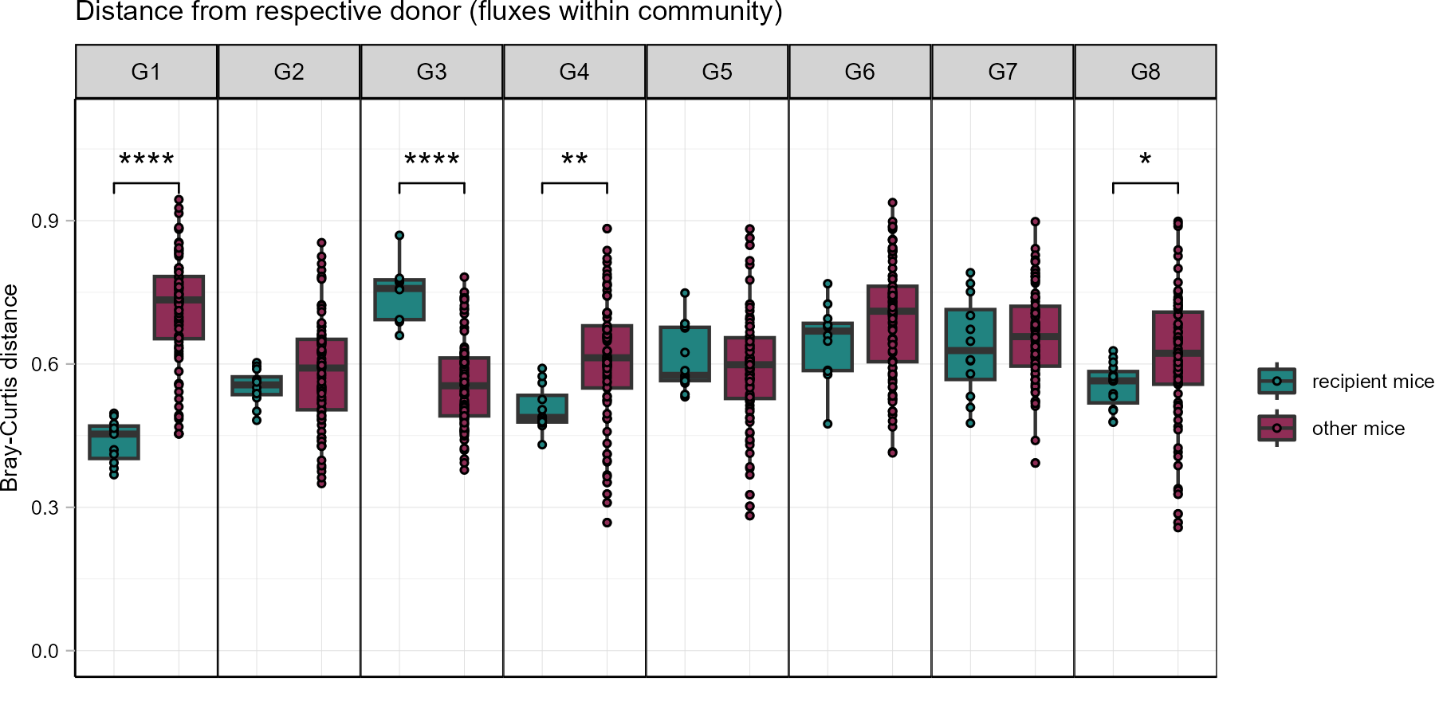


Supplementary Figure 7. Bray-Curtis distance of the predicted metabolic fluxes exchanged in the bacterial community from the donor sample to the respective recipient mice or to all other mice from the remaining seven donor groups, plotted per donor group, all sampling timepoints after gavage included.. BH-corrected Wilcoxon test. (*:p<0.05; **:p<0.01; ***:p<0.001; ****:p<0.0001)


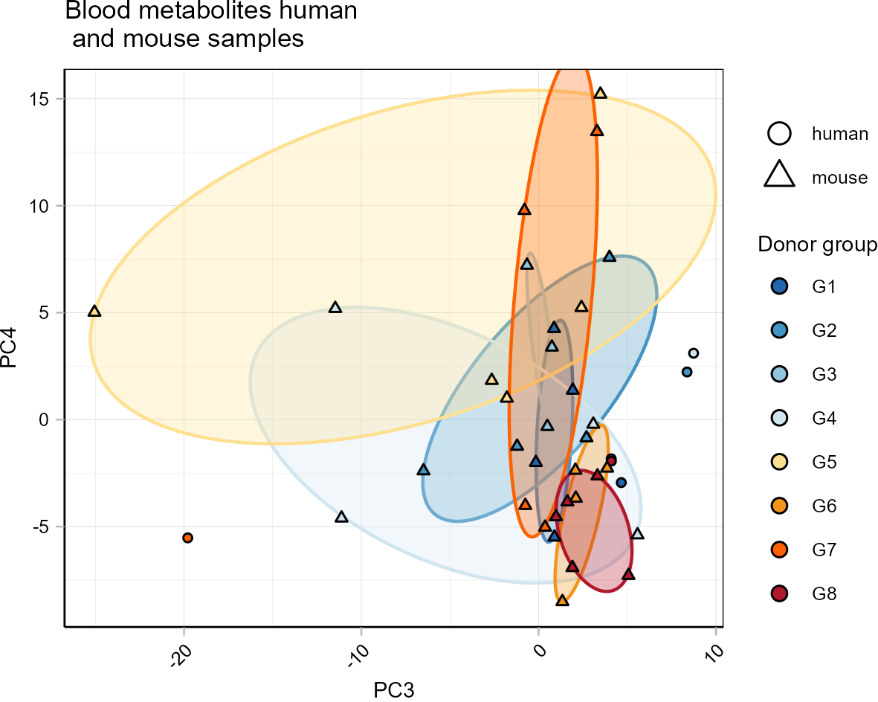


Supplementary Figure 8. Principal component analysis (PCA) of plasma (mice) and serum (human) targeted metabolomics measurements visualising various principal components (PC) to show the differences between species and between donor groups in the mice, PC3 and PC4.
